# High indigestible dietary protein impairs growth and health status of nursery pigs with *Salmonella* Typhimurium more than with enterotoxigenic *Escherichia coli* F4 challenge

**DOI:** 10.1093/jas/skaf451

**Published:** 2025-12-24

**Authors:** Taiwo J Erinle, Marllon J K de Oliveira, John K Htoo, S Maria Mendoza, Jenny-Lee Thomassin, Daniel A Columbus

**Affiliations:** Prairie Swine Centre, Inc, Saskatoon, SK S7H 5N9, Canada; Department of Animal and Poultry Science, University of Saskatchewan, Saskatoon, SK S7N 5A8, Canada; Prairie Swine Centre, Inc, Saskatoon, SK S7H 5N9, Canada; Evonik Operations GmbH, 63457, Germany; Evonik Corporation, Kennesaw, GA 30144; Department of Biochemistry, Microbiology and Immunology, University of Saskatchewan, Saskatoon, SK S7N 5E5, Canada; Prairie Swine Centre, Inc, Saskatoon, SK S7H 5N9, Canada; Department of Animal and Poultry Science, University of Saskatchewan, Saskatoon, SK S7N 5A8, Canada

**Keywords:** acute-phase protein, antioxidant status, enteric pathogenic challenge, post-weaning, standardized ileal digestible crude protein, total crude protein

## Abstract

Indigestible dietary protein content is an emerging dietary concept that is thought to be related to adverse intestinal health outcomes and increased incidence of pathogen-related diarrhea in pigs. The objective of this study was to examine the effect of IDP on growth performance, immune status, and fecal consistency score (**FCS**) of nursery pigs challenged with enterotoxigenic *Escherichia coli* F4 (**ETEC**) or *Salmonella* Typhimurium (**ST**). Thirty-two mixed-sex nursery pigs with an average initial body weight of 7.26 ± 0.40 kg were individually housed and randomly assigned to 1 of 2 dietary treatments for 14 d in a completely randomized design (*n* = 8 pigs/treatment). Corn-soybean-based diets were formulated to contain similar dietary protein (**DP**) content (21%) but differed in IDP content [low IDP, 2.74% (**LIDP**) or high IDP, 4.2% (**HIDP**)]. After a 7-d pre-inoculation period, all the pigs were orally inoculated with either 1.36 × 10^6^ CFU·mL^−1^ ETEC or 1.14 × 10^10^ CFU·mL^−1^ ST (*n* = 16 pigs/challenge). Growth performance, rectal temperature, FCS, and blood inflammatory biomarkers were measured pre- and post-inoculation. There was no dietary effect on any measures pre-inoculation. Inoculation with ETEC or ST increased rectal temperature, FCS, blood inflammatory cytokines, acute-phase protein, and redox biomarkers (*P *< 0.05). Unlike in ETEC-inoculated pigs (*P *> 0.05), HIDP decreased average daily feed intake and average daily gain (*P *< 0.05) and tended to decrease gain: feed (*P *< 0.10) compared to LIDP in ST-inoculated pigs. Low IDP reduced FCS (*P *< 0.05) and tended to reduce ileal and cecal ammonia-nitrogen concentrations (*P *< 0.10) in ST-pigs compared to HIDP but not in ETEC-inoculated pigs. Regardless of enteric pathogen, HIDP stimulated immune response with higher serum interleukin-6 and plasma haptoglobin compared to LIDP (*P *< 0.05). In ST-inoculated pigs, HIDP further increased serum albumin, tumor necrotic factor-alpha, and diamine oxidase, pathogen translocation to mesenteric lymph node, while reducing plasma reduced glutathione (**GSH**) and GSH: oxidized glutathione (*P *< 0.05). Increasing IDP content results in negative outcomes on performance, fecal score, and inflammation in ST-inoculated pigs, with milder outcomes under ETEC challenge.

## Teaser

Rather than varying dietary protein level, the negative effects of undigested protein and its fermentation on hindgut health of weaning pigs may be better understood by determining indigestible protein content, particularly when pigs are exposed to enteric pathogens. The results of the present study provide evidence of interactive effects of indigestible protein content and pathogen-type on performance and gut health of nursery pigs.

## Introduction

Among the myriad of stressors piglets face during weaning, diet transition and pathogenic exposure are significant contributory factors causing post-weaning diarrhea (**PWD**) (Upadhaya and Kim, 2021; Soliani et al., 2023; Jin et al., 2024). Enterotoxigenic *Escherichia coli* F4 (**ETEC**) and *Salmonella enterica* subsp. *enterica* serotype Typhimurium (**ST**) are among the identified pathogens implicated in the pathogenesis of PWD (Zhang et al., 2003; García et al., 2020; Li et al., 2024) and gastroenteritis in pigs. While both pathogens belong to the family *Enterobacteriaceae*, they differ in their mechanisms of action, virulence, and tropism for specific tissue in the gastrointestinal tract (**GIT**). Following infection, ETEC that survives the stomach acid transits to the upper GIT (jejunum and ileum), where fimbriae are used to adhere and colonize the host epithelium. Subsequently, heat-labile enterotoxin and/or heat-stable enterotoxins are produced and secreted, which induces diarrhea in the host (Fleckenstein et al., 2010; Luppi, 2017; Mueller and Tainter, 2023). Meanwhile, ST transits through the small intestine, where it adheres to and colonizes the distal GIT (ileum, cecum, and colon), then proceeds to invade the intestinal epithelium by outcompeting intestinal commensal microbes and evoke virulent action using specialized type VI and III secretion systems (Müller et al., 2012; Jørgensen et al., 2013; Bao et al., 2020). Regardless of their infection mechanisms, ETEC and ST have been shown to induce intestinal inflammation and alter oxidative stress status (Drumo et al., 2015; Jin et al., 2024). Given the differential GIT section where ETEC or ST exerts their virulence, evaluating their impacts in nursery pigs fed diets with a regional effect would be an exciting inquiry.

One factor thought to have a significant impact on the incidence of diarrhea in post-weaned pigs is dietary protein (**DP**). High-protein diets, ranging from ≥21% DP, are thought to predispose pigs to PWD by increasing undigested protein available for hindgut microbial fermentation (Fang et al., 2019; Zhang et al., 2020; Rocha et al., 2022). In mice, feeding diet containing 54% DP content was shown to alter gut microbial community structure and increase intestinal inflammation and disrupt intestinal barrier functions (Snelson et al., 2021). Consequently, a recent approach is aimed toward reducing the amount of fermentable protein in the distal GIT by lowering total dietary DP in pigs (Li et al., 2019; Luise et al., 2021; Liu et al., 2022). While there is a tendency for reduced PWD incidence and severity by lowering total DP (Marchetti et al., 2023), this effect is not consistent across studies when examining diets with similar DP (Zhang and Piao, 2022) or varying DP content (Rodrigues et al., 2021; Pearce et al., 2024). This suggests that an additional dietary factor other than total DP is involved. It has been suggested that determining the indigestible protein (**IDP**) fraction of a diet may be a better indicator of potential negative effects of a diet. Our recent meta-analysis showed that the IDP fraction was a more relevant dietary factor influencing weaning outcomes, particularly performance response in piglets, and that the fraction increases with increasing inclusion of plant-based protein sources (de Oliveira et al., 2025). We have previously examined the impact of IDP in healthy nursery pigs (Erinle et al., 2025a), however, the impact of IDP in pathogen-challenged pigs and the effect of pathogen type have yet to be examined in nursery pigs. The objective of the present study was to determine the effects of IDP content on growth performance, blood redox and immune status, and infection outcomes of nursery pigs challenged with ETEC or ST. It was hypothesized that greater IDP content in nursery diet would negatively impact growth response, diarrhea, and immuno-oxidative status of piglets of piglets under enteric pathogenic challenge.

## Materials and Methods

### Ethics statement

The experimental protocol (#20230020) was approved by the University of Saskatchewan’s Animal Research Ethics Board. The pigs were handled following the established guidelines by the Canadian Council on Animal Care (CCAC, 2009).

### ETEC susceptibility testing and piglet selection for ETEC challenge

At 3 d old, piglets were tail-docked, and tail samples were collected and stored at −20°C until analysis. Presence of a MUC4 polymorphism, encoding the ETEC receptor gene, in the tail samples was determined following a DNA marker-based test described by Jensen et al. (2006). Briefly, DNA in the tail (approximately 25 mg or 0.5 cm from the tail tip) was extracted by heating each tail sample to 95°C in 200 μl of solution (1.25 ml of 25 mM sodium hydroxide and 10 μL of 0.2 mM ethylene diamine tetraacetic acid) for 60 min, followed by the addition of 200 μl of 40 mM tris-hydrochloric acid. The mixture was then vortexed and centrifuged at high speed for 10 min to obtain the extracted DNA. Subsequently, PCR assays were performed on each sample using 2 µL of extracted DNA sample, 0.5 µL of 10 mM dNTP, 0.2 µL of DreamTaq DNA polymerase (Catalogue # FEREP0701; Fisher Scientific, Canada), 1 µL of 10 µM of primers: 5′GTGCCTTGGGTGAGA GGTTA-3′/5′-CACTCTGCCGTTCTCTTTCC-3′ (Integrated DNA Technologies, Inc., Canada), 2.5 µL of 10X buffer, and 17.8 µL of nuclease-free water. Thermocycling was performed using 3 min initial denaturation at 95 °C, followed by 95°C for 30 s, annealing temperature was 65°C for 30 s, and extension at 72°C for 10 min for 35 cycles. The resultant PCR product was 367 bp. Furthermore, 5 µL of each PCR product was treated using Xbal (Catalogue # FERFD0684; Fisher Scientific, Canada) at 37°C for 15 min according to the manufacturer’s recommendation. Following restriction enzyme treatment, 15 µL of each sample was separated by electrophoresis on a 2% agarose gel in 1X TAE buffer (40 mM Tris–acetate [pH 7.4], 20 mM sodium acetate, 1 mM disodium EDTA) containing ethidium bromide DNA stain at 100 V for 20 min. Electrophoresed DNA was then visualized using a ChemiDoc MP Imaging System. PCR amplicons from pig tails containing the resistant allele (R) were not cleaved by XbaI; whereas, those containing susceptible allele (S) were cleaved resulting in the presence of DNA products at 151 and 216 bp. In this study, we observed a susceptibility rate of 36.5% among the total pigs tested. Pigs (*n* = 16) with allele S and identical BW were selected for the ETEC challenge study.

### Animals, housing, diets, and experimental design

A total of 32 mixed-sex, gender-balanced newly weaned pigs (Camborough Plus × C3378; PIC Canada Limited; 22 ± 1 days old; 7.3 ± 0.1 kg body weight [**BW**]) were obtained from the Prairie Swine Centre, Inc. (Saskatoon, SK, Canada) and transported to the Biosafety Level 2, Animal Care Unit of the Western College of Veterinary Medicine (Saskatoon, SK, Canada). Two days before weaning, pigs were vaccinated with 1 mL porcine circovirus vaccine (Ingelvac CircoFLEX^®^; Boehringer Ingelheim). Pigs were housed individually on solid floors with rubber mats in a temperature-controlled room (26°C and decreased by 0.5°C weekly) and fixed photoperiod of 12 h, provided by artificial light. Pigs were randomly assigned to 1 of 2 dietary treatments (low [**LIDP**] or high IDP [**HIDP**]) and then to 1 of 2 enteric pathogenic challenges (ETEC or ST at 16 pigs/pathogen) in a randomized complete block randomized design (n = 8 pigs/treatment) for 14 d, comprising 7-d pre-inoculation (adaptation) and 7-d post-inoculation periods over two blocks (n = 16 pigs/block/pathogen). Dietary treatments were corn-soybean-based and contained similar dietary protein (**DP**) content (21%), but different levels of IDP (LIDP [2.74%] or HIDP [4.20%]) (Tables 1 and 2), as previously reported by Erinle et al. (2025a). The IDP levels were mathematically estimated as given below:IDP (%) = Total DP (%) − Standardized ileal digestible DP (%)

**Table 1.** Ingredient and nutrient compositions of experimental diets (as-fed basis, %, unless otherwise stated)^1^

	Diets^1^	
Ingredients, %	LIDP	HIDP
**Corn**	59.80	36.49
**Soybean meal**	9.61	9.61
**Soybean protein concentrate**	15.55	3.76
**Corn DDGS**	–	19.46
**Whey permeate**	10.00	10.00
**Canola oil**	0.56	4.46
**Canola meal**	–	12.00
**L-Lys HCl**	0.50	0.76
**DL-Methionine**	0.29	0.23
**L-Threonine**	0.21	0.24
**L-Tryptophan**	0.10	0.12
**L-Isoleucine**	0.13	0.07
**L-Valine**	0.13	0.13
**Limestone**	1.30	1.41
**Monocalcium phosphate**	1.27	0.71
**Salt**	0.40	0.40
**Vitamin-mineral premix^2^**	0.15	0.15
***Calculated nutrient content* ^3^**		
**Dry matter, %**	89.1	87.0
**Net energy, kcal/kg**	2500	2500
**Crude protein, %**	21.0	22.0
**SID crude protein, %**	18.3	17.8
**IDP, %**	2.74	4.20
**Crude fiber, %**	2.15	4.07
**Total DF, %**	10.4	15.7
**Insoluble DF, %**	9.63	14.9
**Soluble DF, %**	0.72	0.85
**Insoluble DF: Soluble DF**	13.4	17.5
**Soluble DF: Total DF, %**	0.07	0.05
**Insoluble DF: Total DF, %**	0.93	0.95
**Calcium,%**	0.80	0.80
**Phosphorus, %**	0.62	0.68
**STTD phosphorus, %**	0.40	0.40
**Calcium: STTD Phosphorus**	2.00	2.00
***Amino acids, % SID***		
**Arginine**	1.23	1.04
**Histidine**	0.46	0.45
**Isoleucine**	0.89	0.74
**Leucine**	1.55	1.57
**Lysine**	1.35	1.35
**Methionine**	0.55	0.52
**Cysteine**	0.26	0.29
**Methionine + cysteine**	0.81	0.81
**Phenylalanine**	0.89	0.81
**Tyrosine**	0.58	0.52
**Phenylalanine + tyrosine**	1.47	1.34
**Threonine**	0.85	0.85
**Tryptophan**	0.30	0.30
**Valine**	0.96	0.92

DF, dietary fiber; IDP, indigestible dietary protein; SID, standardized ileal digestible; STTD, standardized total tract digestible.

1 Experimental diets with low IDP (LIDP) and high IDP (HIDP).

2 Supplied per kg of complete diet: vitamin A, 6,000 IU; vitamin D, 9.3 mg; vitamin E, 35 IU; menadione, 2.5 mg; vitamin B12, 0.02 mg; thiamine, 1.00 mg; biotin, 0.10 mg; niacin, 20 mg; riboflavin, 4 mg; pantothenate, 12 mg; folic acid, 0.50 mg; pyridoxine, 5.0 mg; Fe, 75 mg; Zn, 75 mg; Mg, 20 mg; Cu, 10 mg; Se, 0.15 mg, and I, 0.50 mg.

3 Nutrient content of diets based on estimated nutrient contents of ingredients according to NRC (2012) and AminoDat 5.0  (Evonik, 2016)  and analyzed amino acid content by AMINOLab of Evonik Operations GmbH.

**Table 2.** Analyzed nutrient content of experimental diets (as-fed basis, %, unless otherwise stated)

	Diets^1^	
Item	LIDP	HIDP
**Dry matter, %**	89.09	89.6
**Gross energy, kcal/kg**	3914	4133
**Crude protein, %**	20.0	20.9
***Total amino acids* ^2^**		
**Methionine**	0.54 (0.58)	0.54 (0.57)
**Cystine**	0.32 (0.31)	0.39 (0.37)
**Methionine + cystine**	0.86 (0.89)	0.93 (0.94)
**Lysine**	1.44 (1.46)	1.51 (1.54)
**Threonine**	0.94 (0.96)	1.00 (1.02)
**Tryptophan**	0.31 (0.33)	0.33 (0.34)
**Arginine**	1.27 (1.30)	1.15 (1.17)
**Isoleucine**	0.96 (0.97)	0.87 (0.87)
**Leucine**	1.67 (1.72)	1.82 (1.83)
**Valine**	1.07 (1.07)	1.11 (1.10)
**Histidine**	0.51 (0.52)	0.53 (0.54)
**Phenylalanine**	0.97 (0.98)	0.95 (0.96)
**Glycine**	0.82	0.87
**Serine**	0.97	0.95
**Proline**	1.13	1.36
**Alanine**	0.99	1.12
**Aspartic acid**	1.99	1.67
**Glutamic acid**	3.39	3.48

DF, dietary fiber; IDP, indigestible dietary protein; SID, standardized ileal digestible.

1 Experimental diets with low IDP (LIDP) and high IDP (HIDP).

2 Analyzed values of individual amino acids with corresponding calculated values in brackets.

The IDP levels were based on the difference between DP and SID DP and were achieved using protein sources that were digestible (soy protein concentrate) and less digestible (canola meal and corn distiller dried grain with solubles). Diets were formulated to meet or exceed nutrient requirements for 7–11 kg pigs according to NRC (2012) and AMINODat 6.0 and using published nutrient content of ingredients according to NRC (2012) and AMINODat 6.0 (Evonik, 2016). Amino acid content of major protein ingredients (e.g., soy protein concentrate, soybean meal, canola meal, and corn DDGS) was analyzed prior to formulation (Evonik Corporation, Kennesaw, GA). Pigs were given unrestricted access to both feed and water.

### Pathogens, culture condition, and oral inoculation

#### *Enterotoxigenic* E. coli *F4*

The ETEC containing enterotoxins, including estA (heat-stable toxins, STa and STb), elt (heat-labile toxin), and faeG (adhesion F4 fimbriae), was obtained from Dr Chengbo Yang’s Laboratory, University of Manitoba, Manitoba, Canada, and was selected for antibiotic resistance to chloramphenicol. The ETEC culture was prepared following a procedure adapted from Jayaraman et al. (2017). In brief, from a glycerol stock, ETEC was streaked on Luria-Bertani agar Miller (**LB**; BP1425-500, Fisher Scientific, Canada) and incubated overnight (**O/N**) for 16–18 h at 37 °C. A single colony was transferred into glass tubes containing 5 mL tryptic soy broth (**TSB**; BD Bacto, Fisher Scientific, Canada), inclined at 45^°^, and incubated 16–18 h at 37 °C with aeration (200 rpm). A total of 200 μL of the resultant inoculum was added into a flask containing 10 mL TSB, incubated at 37°C with aeration (200 rpm) for 1–2 h until the bacteria reached an optical density (**OD**) at 595 nm of 0.45–0.50 (UVD spectrophotometer 144509, Helios Delta, Thermo Electron Corporation, USA), which was previously determined to be exponential phase. The ETEC was then diluted to a final concentration of 1.36 × 10^6^ colony forming units (**CFU**)·mL^−1^ in phosphate buffered saline (**PBS**; pH 7.4). Inoculum concentrations were verified by serial dilution and plated onto LB agar followed by colony forming enumeration the next day. On day 0 pre-inoculation, the inoculum was transported on ice to the experimental room and pigs (n = 16) were orally inoculated with 5 mL ETEC (final concentration 1.36 × 10^6^ CFU·mL^−1^) at 0900 h following the procedure described by Choi et al. (2020).

#### Salmonella *Typhimurium var. Copenhagen*

*Salmonella* Typhimurium var. Copenhagen selected for antibiotic resistance to nalidixic acid (**Nal^+^**) and novobiocin (**Nov^+^**), was cultured according to Wellington et al. (2019). Briefly, from a glycerol stock, ST was streaked purified onto a brilliant green (**BG**) agar plate containing 30 µg/mL Nal^+^/50 µg/mL Nov^+^ and incubated O/N at 37°C. A single colony was transferred into an Erlenmeyer flask containing 15 mL LB and incubated at 37°C with aeration (200 rpm) for 16–18 h. The next day, bacteria were subcultured 1/100 into fresh LB and incubated at 37°C with aeration (200 rpm) for 1.5 h until the bacteria reached an OD_595_ of 0.65–0.75 (UVD spectrophotometer 144509, Helios Delta, Thermo Electron Corporation, USA), which is equivalent to 2 × 10^8^ CFU·mL^−1^. The ST were collected by centrifugation for 15 min at 5,730×*g* at 4 °C, culture supernatants discarded, then ST were resuspended in a volume of PBS needed to achieve a final ST inoculum concentration of 1.14 × 10^10^ CFU·mL^−1^). Inoculum concentrations were verified by serial dilution, spot plating onto BG agar containing 30 µg/mL Nal^+^/50 µg/mL Nov^+^, followed by colony forming enumeration the next day. On day 7 pre-inoculation, the inoculum was transported on ice to the experimental room and pigs (n = 16) were orally inoculated twice within 4 h with 1 mL ST (final concentration 1.14 × 10^10^ CFU·mL^−1^) at 0900 h.

#### Growth performance

On day 7 pre-inoculation, day 0, and day 7 post-inoculation, BW of pigs and their feed intake were individually measured. The individual BW and feed intake were used to determine the average daily gain (**ADG**), average daily feed intake (**ADFI**), and feed efficiency (gain:feed; **G:F**) of pigs during the pre- and post-inoculation.

#### Sample collection

On day 1 pre-inoculation and 8 h (ETEC) days 1, 2, 3, 4, 5, and 6 post-inoculation, fecal samples and core body temperature measurements via the rectum were collected from all pigs. The feces were visually scored for consistency score. In ETEC-inoculated pigs, fecal consistency score (**FCS**; 0. normal well-formed solid feces; 1. formed soft feces; 2. mild diarrhea (i.e., fluid feces with yellowish color); 3. severe diarrhea (i.e., watery and projectile feces) were recorded as described by Choi et al. (2020). In ST-inoculated pigs, FCS (0, normal consistency; 1, semi-solid feces without blood; 2, watery feces without blood; and 3, blood-tinged feces) was recorded as described by Rodrigues et al. (2021). In addition, fecal samples collected on day 1 pre-inoculation, day 4 and 7 post-inoculation were snap-frozen in liquid nitrogen and later stored at −80°C until further analysis. Also, on day 1 pre-inoculation, day 4 and 7 post-inoculation, blood samples were also collected from all pigs at 0900 h in 10 mL sodium-heparin coated vacutainers (BD, Mississauga, ON Canada) and anticoagulant-free vacutainers via jugular vein puncture. Blood collected in vacutainers with no anticoagulant was allowed to clot. Subsequently, all blood samples were centrifuged at 2,500×*g* for 15 min at 4°C to obtain plasma and serum. The plasma and serum samples were aliquoted into 2 mL microtubes and stored at −20°C until further analyses. On day 7 post-inoculation, all pigs were humanely euthanized using a penetrative captive bolt for the collection of samples, including feces, digesta (from the distal ileum and mid-section of cecum and colon), and tissue (ileal mesenteric lymph node (**MLN**), liver, and spleen). The samples were stored appropriately until required for further analysis.

#### Digesta and tissue analyses

Digesta samples (approximately 1 g) from the ileum, cecum, and colon were aseptically collected into sterile tubes containing 4 mL buffered peptone water (**BPW**) and homogenized. The digesta samples were sequentially serially diluted ten-fold (10^−1^) to a final dilution factor of 10^−7^. Subsequently, 200 μL of each serial dilution was plated on MacConkey (**MAC**) agar containing 25 µg/mL chloramphenicol for ETEC-pigs or BG agar plates containing 30 µg/mL Nal^+^/50 µg/mL Nov^+^ for ST-pigs and incubated for 24 h at 37°C. Agar plates containing between 30 and 300 bacterial colonies were enumerated and colony counts were used to calculate CFU/g of ETEC of ST in digesta.

The ammonia-nitrogen (**NH_3_-N**) concentration in the ileal, cecal, and colonic digesta was measured spectrophotometrically based on the reaction of ammonium ions with sodium phenate, sodium nitroprusside, and hypochlorite reagents (Rhine et al., 1998), following a procedure previously described by Erinle et al. (2025b). Briefly, colonic digesta samples were diluted with double-distilled H_2_O at a ratio of 1:1. Approximately 1 g of ileal, cecal, and diluted colonic digesta were centrifuged at 14,000×*g* for 10 min, and 20 µL of supernatant was transferred into glass tubes and covered with parafilm to prevent absorption of ammonia from the air. The standard solution was prepared by dissolving 0.4176 g ammonium sulfate (previously dried at 60°C for 2 h) in 1 L of deionized water. Seven gradient levels of standard solution (0, 10, 20, 30, 40, 50, and 60 μL) were added into glass tubes for standard curve generation. Subsequently, 2 mL of sodium phenate, 3 mL of 0.01% sodium nitroprusside, and 3 mL of 0.02 N hypochlorite working solution were added into each tube containing standard or samples, covered with parafilm and incubated on a shaker for 1 h in the dark for color development. The color development, indicated by increased absorbance, was measured at 600 nm using a spectrophotometer (Model# 4001/4, Genesys 20; Thermo Scientific, USA).

To determine ETEC or ST translocation, 1 g of MLN, liver, and spleen were collected aseptically into sterile tubes containing 20 mL BPW and homogenized. Subsequently, 200 μL of the homogenized mixture was plated on MAC agar containing 25 µg/mL chloramphenicol for ETEC infected-pigs or BG agar plates containing 30 µg/mL Nal^+^/50 µg/mL Nov^+^ for ST infected-pigs. In addition, 1 mL of homogenized mixture was enriched with 4 mL of EC broth containing 25 µg/mL chloramphenicol for ETEC infected-pigs or 4 mL of selenite-cysteine broth containing 30 µg/mL Nal^+^/50 µg/mL Nov^+^ for ST infected-pigs and incubated O/N at 37°C with aeration (200 rpm). Following this, 200 μL of the O/N culture was plated on MAC agar (containing 25 µg/mL chloramphenicol) or BG agar (containing 30 µg/mL Nal^+^/50 µg/mL Nov^+^) and incubated O/N at 37°C. Following incubation, ETEC and ST colonies were enumerated and scored using a modified shedding scoring system adapted from Wellington et al. (2019). Briefly, translocation score of 0 was given to plates without colonies after direct plating and remained negative after O/N enrichment. A translocation score of 1 was given to plates without colonies after direct plating but were positive after O/N enrichment. Plates with <30 colonies of antibiotic-resistant ETEC or ST after direct plating were given a translocation score of 2. Plates with >30 but <300 colonies of antibiotic-resistant ETEC or ST after direct plating were given a translocation score of 3. Plates with >300 colonies of antibiotic resistant ETEC or ST after direct plating were assigned a translocation of score 4.

#### Blood analysis

Serum albumin (**ALB**) was analyzed at Prairie Diagnostic Services (Saskatoon, SK, Canada). Serum samples were analyzed for interleukin-6 (**IL-6**, ab100755; Abcam, Cambridge, MA), diamine oxidase (**DAO**, EK751018; AFG Scientific, USA), and tumor necrotic factor alpha (**TNF-α**, ab100756; Abcam) according to manufacturers’ instructions. Plasma content of haptoglobin (ab205091; Abcam), superoxide dismutase (**SOD**; Item No: 706002, Cayman Chemical, Ann Arbor MI), malondialdehyde (**MDA**, ab118970; Abcam), d-Lactate (ab83429; Abcam), urea nitrogen (**PUN**, ab83362; Abcam), alkaline phosphatase (**ALP**, DALP-250; Gentaur, Bioassay Systems, Hayward, CA, USA), reduced glutathione (**GSH**), oxidized glutathione (**GSSG**), and GSH: GSSG (ab138881; Abcam) were analyzed following the manufacturer’s instructions of the respective kits. For colorimetric or fluorometric assays, absorbances were measured using a colorimetric plate reader (EPOCH2C; BioTek Instruments, Inc. USA) or fluorometric plate reader (S1LF; BioTek Instruments, Inc. USA), respectively.

#### Fecal and digesta myeloperoxidase activity

Feces collected on day 1 pre-inoculation, and days 4 and 7 post-inoculation were analyzed for myeloperoxidase (**MPO**) using a colorimetric enzyme-linked immunosorbent assay kit (ab105136; Abcam, Cambridge, MA) according to manufacturer’s instructions and a modified sample processing procedure reported. Briefly, fecal samples were allowed to thaw at room temperature, thoroughly mixed on ice, and subsampled into 2 mL microtubes. The fecal samples were diluted at 1:1 with double-distilled H_2_O, centrifuged at 6,000×*g* for 20 min at 4°C, and 750 µL of the resultant supernatant was transferred into 2 mL microtubes and centrifuged again at 7,000×*g* for 10 min at 4°C. Following the manufacturer’s instructions, the resultant supernatant was then used for the MPO analysis. Also, approximately 2 g of ileal, cecal, and colonic digesta were collected into 2 mL microtubes and stored at −80°C until required for hindgut NH_3_-N analysis, while ileal and colonic digesta were analyzed for MPO activity following the procedure for fecal MPO described above.

#### Statistical analysis

Normality test of datasets was performed using the PROC UNIVARIATE model and verified using the Shapiro-Wilk test in SAS 9.4 (SAS Institute, Inc. Cary, NC, USA) and outliers were identified and removed where the residual of error terms was ±3 standard deviation from the mean. For all parameters, individual piglets were considered as experimental units. The model analyzed FCS as repeated measures using PROC GLIMMIX with a logistic (binomial) link function, while considering dietary treatment, day, and their interaction as fixed effects, and a random intercept for each pigs. Rectal temperature, fecal MPO, and blood parameters were also analyzed as repeated measures in a complete randomized design (PROC MIXED), including 1) dietary treatments, 2) day, and 3) their interactions. These main effects and their interactions were considered fixed. Mean separation was determined using the Tukey-Kramer test with a significant level set at *P *≤ 0.05. A tendency toward significance was considered at 0.05 < *P *< 0.10.

## Results

### Rectal temperature and fecal consistency score

**Figure 1.**
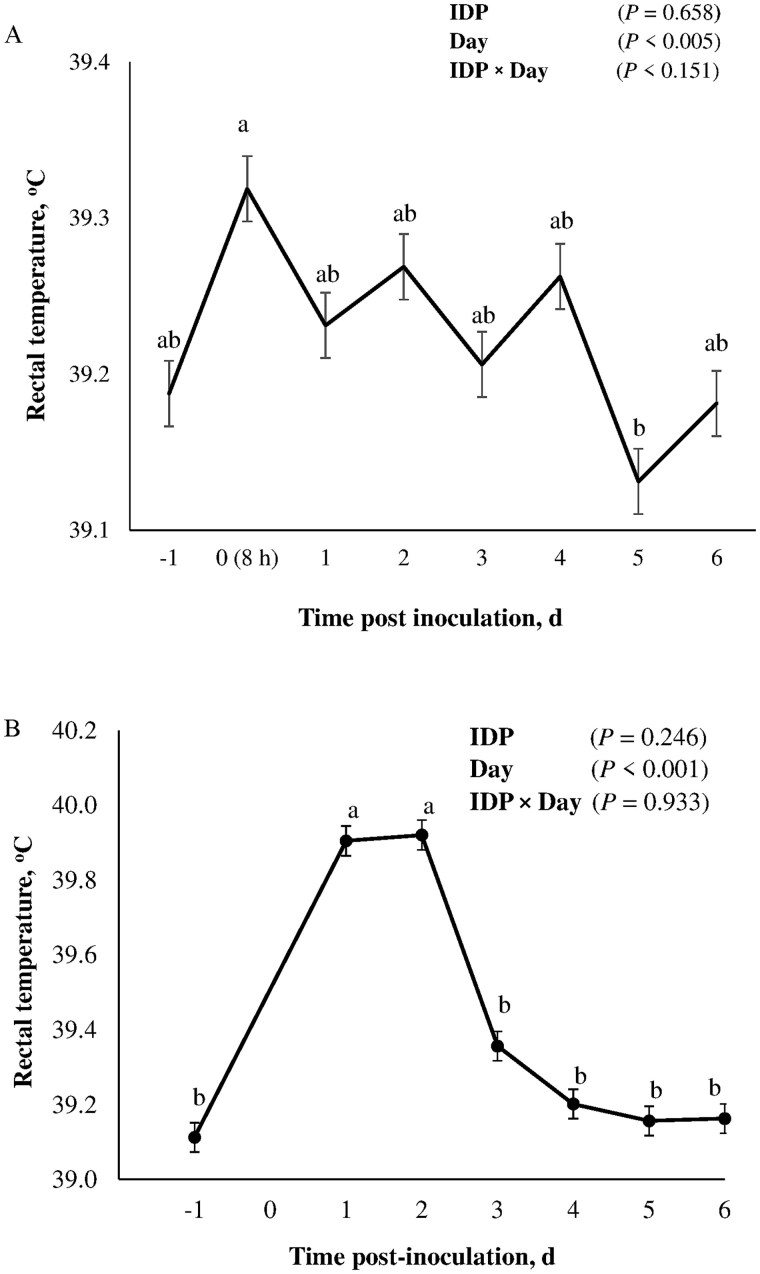
Rectal temperature of piglets fed low (LIDP) or high indigestible dietary protein (HIDP) content and inoculated with enterotoxigenic *E. coli* (ETEC) (A) or *Salmonella* Typhimurium (ST) (B). Within days (day −1 pre-inoculation and 8 h (ETEC), days 1, 2, 3, 4, 5, 6 post-inoculation), points with different superscripts differ (*P *< 0.05). In both ETEC- and ST-inoculated pigs, there was no significant (*P *> 0.10) effect of IDP on rectal temperature, therefore, only time data points are shown. Values are average rectal temperature taken from all pigs per time-point, while the error bars represent the standard error of mean; n = 8 pigs/treatment.

**Figure 2.**
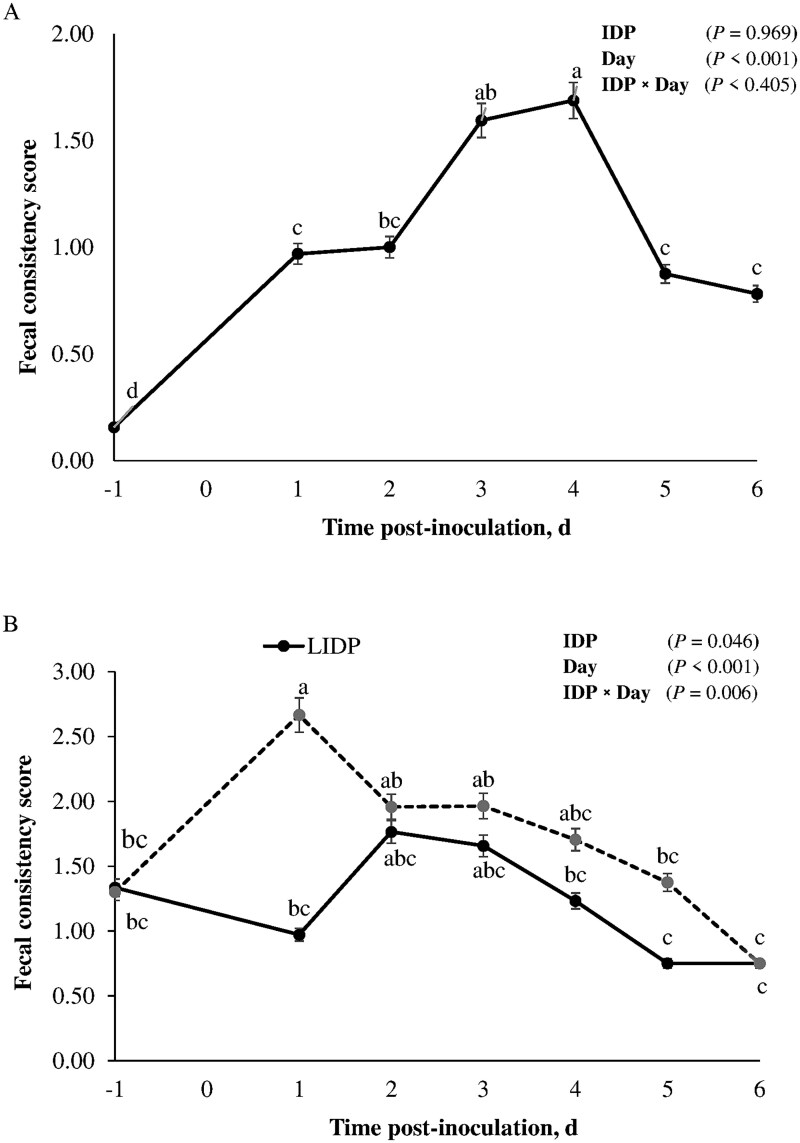
Fecal consistency score (FCS) in piglets fed low (LIDP) or high indigestible dietary protein (HIDP) content and inoculated with enterotoxigenic *E. coli* (ETEC) (A) or *Salmonella* Typhimurium (ST) (B). Within days (day −1 pre-inoculation and day 1, 2, 3, 4, 5, 6 post-inoculation), points with different superscripts differ (*P *< 0.05). In ETEC-inoculated pigs, FCS (0, normal well-formed solid feces; 1, formed soft feces; 2, mild diarrhea (i.e., fluid feces with yellowish color); 3, severe diarrhea (i.e., watery, and projectile feces) were recorded as described by Choi et al. (2020). In ST-inoculated pigs, FCS (0, normal consistency; 1, semi-solid feces without blood; 2, watery feces without blood; and 3, blood-tinged feces) was recorded as described by Rodrigues et al. (2021). There was significant (*P *< 0.05) effect of IDP on FCS of pigs inoculated with ST but not with ETEC, therefore, only time data points are shown for ETEC. Values are least square means ± standard error of the mean; n = 8 pigs/treatment.

Pre- and post-inoculation rectal temperature and FCS of piglets fed LIDP or HIDP diets and challenged with ETEC or ST shown in Figures 1A and B and 2A and B, respectively. In ETEC-inoculated pigs, rectal temperature peaked within 8 h post-inoculation and reached its lowest value on day 5 post-inoculation (*P *< 0.05); however, both values did not differ from the baseline temperature. However, in ST-inoculated pigs, there was an increased in rectal temperature to ≥ 39.9°C within 24 h, which was sustained for 48 h before returning to the baseline temperature (*P *< 0.01). Inoculation with ETEC or ST increased FCS in nursery pigs (*P *< 0.05). Regardless of the pathogenic challenge model, there was no effect of IDP on rectal temperature of pigs (*P *> 0.10). Unlike in ETEC, FCS of pigs challenged with ST was negatively affected by IDP level (*P *= 0.01) and was observed to be higher in pigs fed HIDP compared to LIDP. Unlike in ETEC, there were significant IDP×Day interaction effects on FCS of ST-infected pigs, which became more severe in HIDP-fed pigs compared to the LIDP pigs (*P *< 0.05).

### Growth performance

Growth performance results of piglets fed LIDP or HIDP diet and challenged with ETEC or ST are presented in Table 3. Prior to inoculation with either ETEC or ST, there was no IDP effect on both initial and inoculation BW of pigs (*P *> 0.10). Similarly, there were no IDP or interaction effects on ADFI, ADG, and G:F during the ETEC pre-inoculation period (*P *> 0.05). During ST pre-inoculation periods, ADFI tended to be higher among pigs fed LIDP compared to HIDP of pigs (*P *= 0.07), while ADG and G:F were not affected (*P *> 0.10). During ETEC post-inoculation periods, there was no IDP on growth performance (*P *> 0.10). During ST post-inoculation periods, ADFI and ADG were higher in pigs fed LIDP compared to HIDP (P < 0.05). Unlike in ETEC pigs, there was an IDP effect on final BW of pigs challenged with ST and was observed to be higher among LIDP-fed pigs than those fed HIDP (*P *< 0.05).

**Table 3.** Growth performance of piglets fed low or high indigestible dietary protein and challenged with enterotoxigenic *E. coli* or *Salmonella* Typhimurium

	IDP^1^			*P*-value	
Parameter	LIDP	HIDP	SEM	IDP	
**Enterotoxigenic *E. coli***					
**Initial BW (day** −**7), kg**	7.19	7.24	0.15	0.818	
**Inoculation BW (day 0), kg**	9.08	9.05	0.23	0.922	
**Final BW (day 7), kg**	11.7	11.5	0.38	0.786	
**Pre-inoculation period (day** −**7 to 0)**					
**ADFI, kg**	0.33	0.31	0.03	0.648	
**ADG, kg**	0.27	0.26	0.03	0.810	
**Gain: Feed, kg/kg**	0.80	0.83	0.04	0.609	
**Post-inoculation period (day 0 to 7)**					
**ADFI, kg**	0.59	0.57	0.03	0.646	
**ADG, kg**	0.37	0.35	0.03	0.694	
**Gain: Feed, kg/kg**	0.62	0.61	0.03	0.780	
***Salmonella* Typhimurium**					
**Initial BW (day** −**7), kg**	7.27	7.33	0.10	0.652	
**Inoculation BW (day 0), kg**	8.96	8.59	0.24	0.295	
**Final BW (day 7), kg**	11.3^a,b^	9.92^a,b^	0.37	0.020	
**Pre-inoculation period (day** −**7 to 0)**					
**ADFI, kg**	0.28	0.23	0.02	0.072	
**ADG, kg**	0.24	0.18	0.03	0.110	
**Gain: Feed, kg/kg**	0.83	0.80	0.04	0.670	
**Post-inoculation period (day 0 to 7)**					
**ADFI, kg**	0.50^a,b^	0.37^a,b^	0.04	0.039	
**ADG, kg**	0.34^a,b^	0.19^a,b^	0.04	0.016	
**Gain: Feed, kg/kg**	0.68	0.55	0.05	0.113	

BW, body weight; ADG, average daily gain; ADFI, average daily feed intake; IDP, indigestible dietary protein.

1 Experimental diets with low IDP (LIDP) and high IDP (HIDP). Values are least square means; n = 8 pigs/treatment.

a,b In a row, means assigned different lowercase superscript letters are significantly different (*P *< 0.05).

### ETEC and ST quantification in digesta and translocation into tissue

**Figure 3.**
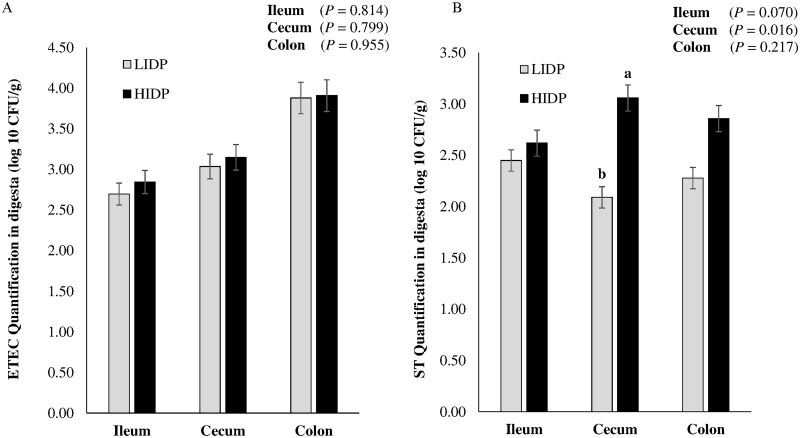
Enterotoxigenic *Escherichia coli* F4 (ETEC) (A) and *Salmonella* Typhimurium var. Copenhagen (ST) (B) quantification in intestinal content (log 10 CFU/g; day 7 post-inoculation) of infected pigs fed low (LIDP) or high indigestible dietary protein (HIDP). Values are least square means ± standard error of the mean; n = 8 pigs/treatment. ^a,b^On a bar, means assigned different lowercase superscript letters are significantly different (*P *< 0.05).

**Figure 4.**
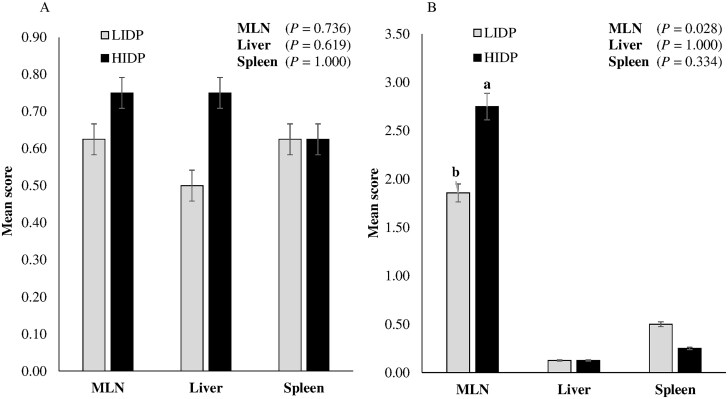
Enterotoxigenic *Escherichia coli* F4 (ETEC) (A) and *Salmonella* Typhimurium var. Copenhagen (ST) (B) translocation to the mesenteric lymph node (MLN), liver, and spleen in pigs fed varying indigestible dietary protein (IDP) content. Values are least square means ± standard error of the mean; n = 8 pigs/treatment. ^a,b^On a bar, means assigned different lowercase superscript letters are significantly different (*P *< 0.05).

The ETEC and ST quantification in intestinal content is shown in Figure 3A and B, respectively, while their translocation to MLN, liver, and spleen is presented in Figure 4A and B, respectively. There was no IDP effect on ETEC quantification score in ileal, cecal, and colonic digesta (*P *> 0.10) and translocation to MLN, liver, and spleen (*P *> 0.10). Unlike in colonic digesta, IDP showed a significant effect on ST quantification in cecal digesta (*P *= 0.02) and tended to be significant in ileal digesta (*P *= 0.07), with quantification values lower in LIDP-fed pigs compared to those fed HIDP. Also, unlike in the liver and spleen, pigs fed LIDP had significantly lower ST translocation to MLN compared to those fed HIDP (*P *= 0.03).

### Blood parameters

Pre- and post-inoculation redox and inflammatory biomarkers and acute-phase protein concentrations in blood of ETEC- and ST-infected pigs are presented in Tables 4 and 5, respectively. Both ETEC and ST inoculation increased serum ALB (*P *< 0.05), which was higher on day 4 post-inoculation and decreased on day 7 (*P *< 0.01). Unlike in ETEC-inoculated pigs, serum ALB was higher in ST-inoculated pigs fed HIDP compared to LIDP (*P *= 0.008). Regardless of the pathogen type, ALB was higher in male piglets than in females (*P *< 0.05). In addition, both ETEC and ST inoculation increased serum IL-6 (*P *< 0.05), particularly on day 4 post-inoculation, and was observed to be higher among HIDP-fed pigs. Serum TNF-α tended to be influenced by ETEC inoculation, which was higher on day 4 post-inoculation (*P *= 0.07). Unlike ETEC, serum TNF-α was influenced by ST inoculation, which was higher throughout post-inoculation days (*P *< 0.01). In contrast to the lack of IDP effect in ETEC-pigs (*P *> 0.10), serum TNF-α in ST-inoculated pigs was higher among HIDP pigs compared to those fed LIDP (*P *= 0.01). Serum DAO was increased by ETEC or ST inoculation mostly on day 4 post-inoculation (*P *< 0.05). In contrast to ETEC-pigs, serum DAO in ST-inoculated pigs was higher among HIDP pigs compared to those fed LIDP (*P *= 0.04). Unlike the tendency in ETEC infected-pigs, ST infection significantly influenced plasma haptoglobin. Pigs fed HIDP had higher or tended to have higher plasma haptoglobin under ST- (*P *= 0.06) or ETEC-inoculation (*P *< 0.01), respectively. Plasma d-Lactate was elevated following ETEC (*P *= 0.02) or ST (*P *= 0.03) inoculation and was observed to be higher on day 4 or 7, respectively. There was no IDP effect on plasma d-Lactate (*P *> 0.10). Unlike in ST-pigs, plasma ALP was significantly influenced by ETEC inoculation and was observed to be higher on day 4 post-inoculation (*P *< 0.01). Plasma SOD and MDA were significantly affected by ETEC and ST inoculation and were observed to be higher on day 4 or 7 of respective pathogenic challenge (*P *< 0.01). Notwithstanding ETEC or ST inoculation, plasma SOD and MDA were not affected by IDP (*P *> 0.10) but were higher in ST-male pigs compared to females (*P *< 0.05). Plasma GSH and GSH: GSSG were reduced following ETEC or ST inoculation (*P *< 0.01) and remained low throughout the experimental period in the former but restored on day 7 post-inoculation in the latter (*P *< 0.01). In ETEC infected-pigs, LIDP tended to increase plasma GSH compared to HIDP (*P *= 0.07). Under ST inoculation, plasma GSH and GSH: GSSG were significantly increased among LIDP-fed pigs compared to HIDP-pigs (*P *< 0.05). Inoculation with ETEC increased plasma GSSG on day 4 post-inoculation, which was later restored on day 7 post-inoculation (*P *< 0.01). In ST-pigs, there was a significant IDP-Day interaction effect on plasma GSSG (*P *= 0.05). In the ETEC group, PUN was higher in LIDP-fed pigs compared to those fed HIDP (*P *< 0.01). In ST-pigs, there was a significant IDP-Day interaction effect on plasma GSSG (*P *= 0.04).

**Table 4.** Pre- and post-inoculation blood parameters of piglets fed low or high indigestible dietary protein contents and challenged with enterotoxigenic *E. coli *^1^

	IDP				*P*-value		
Parameters	LIDP	HIDP	Day	SEM	IDP	Day	IDP × day
**Serum albumin, g/L**							
**Pre-inoculation (day** −**1)**	28.1	29.5	28.8^y^	0.88	0.162	<0.001	0.766
**Post-inoculation (day 4)**	35.3	38	36.6^x^				
**Post-inoculation (day 7)**	27.3	28.5	27.9^y^				
**Serum interleukin-6, pg/mL**							
**Pre-inoculation (day** −**1)**	47.3	53.2	40.2^z^	9.45	0.015	0.017	0.791
**Post-inoculation (day 4)**	74.0	104	162^x^				
**Post-inoculation (day 7)**	48.1	95.1	125^y^				
**Serum TNF-α, ρg/mL**							
**Pre-inoculation (day** −**1)**	334	322	328	12.0	0.680	0.074	0.780
**Post-inoculation (day 4)**	368	377	373				
**Post-inoculation (day 7)**	374	356	365				
**Serum diamine oxidase, mg/mL**							
**Pre-inoculation (day** −**1)**	25.4	26.3	25.9^y^	1.01	0.559	0.002	0.989
**Post-inoculation (day 4)**	32.0	33.1	32.5^x^				
**Post-inoculation (day 7)**	29.3	29.9	29.6^xy^				
**Plasma haptoglobin, ng/mL**							
**Pre-inoculation (day** −**1)**	24.3	29.9	22.1^y^	4.22	0.1	0.031	0.676
**Post-inoculation (day 4)**	35.1	50.2	42.7^x^				
**Post-inoculation (day 7)**	32.0	36.1	34.1^xy^				
**Plasma d-Lactate, nmol/mL**							
**Pre-inoculation (day** −**1)**	9.37	9.14	9.26^y^	1.38	0.234	0.017	0.041
**Post-inoculation (day 4)**	10.4	15.9	13.2^x^				
**Post-inoculation (day 7)**	10.6	9.41	10.0^xy^				
**Plasma alkaline phosphatase, IU/L**							
**Pre-inoculation (day 6)**	83.8	71.9	77.9^y^	4.54	0.430	0.003	0.713
**Post-inoculation (day 4)**	109	104	106^x^				
**Post-inoculation (day 7)**	87.2	88.2	87.7^xy^				
**Plasma superoxide dismutase, U/mL**							
**Pre-inoculation (day** −**1)**	0.13	0.15	0.14^y^	0.16	0.962	0.015	0.460
**Post-inoculation (day 4)**	0.15	0.15	0.15^xy^				
**Post-inoculation (day 7)**	0.20	0.18	0.19^x^				
**Plasma malondialdehyde, nmol/mL**							
**Pre-inoculation (day** −**1)**	0.39	0.43	0.41	0.06	0.467	<0.001	0.987
**Post-inoculation (day 4)**	0.89	0.95	0.92				
**Post-inoculation (day 7)**	0.44	0.51	0.48				
**Reduced glutathione (GSH), µM**							
**Pre-inoculation (day** −**1)**	4.26	3.49	3.88^x^	0.13	0.073	<0.001	0.143
**Post-inoculation (day 4)**	3.28	2.96	3.12^y^				
**Post-inoculation (day 7)**	2.62	2.73	2.67^y^				
**Oxidized glutathione (GSSG), µM**							
**Pre-inoculation (day** −**1)**	0.72	0.73	0.73^y^	0.16	0.625	<0.001	0.864
**Post-inoculation (day 4)**	1.00	1.00	1.00^x^				
**Post-inoculation (day 7)**	0.81	0.85	0.83^y^				
**GSH: GSSG**							
**Pre-inoculation (day** −**1)**	5.42	4.91	5.16^x^	3.85	0.192	<0.001	0.685
**Post-inoculation (day 4)**	3.32	2.99	3.15^y^				
**Post-inoculation (day 7)**	3.26	3.22	3.24^y^				
**Plasma urea nitrogen, nmol/µL**							
**Pre-inoculation (day** −**1)**	184	120	152	12.7	0.001	0.363	0.232
**Post-inoculation (day 4)**	149	126	137				
**Post-inoculation (day 7)**	171	70.5	121				

IDP, indigestible dietary protein; TNF-α, tumor necrosis factor-alpha.

1 Experimental diets with low IDP (LIDP) and high IDP (HIDP). Values are least square means; n = 8 pigs/treatment.

x,y,z In a column, means assigned different lowercase superscript letters are significantly different (*P *< 0.05).

**Table 5.** Pre- and post-inoculation blood parameters of piglets fed low or high indigestible dietary protein contents and challenged with *Salmonella* Typhimurium^1^

	IDP				*P*-value		
Parameters	LIDP	HIDP	Day	SEM	IDP	Day	IDP × day
**Serum albumin, g/L**							
**Pre-inoculation (day** −**1)**	27.5	28.5	28.0^z^	0.50	0.008	<0.001	0.409
**Post-inoculation (day 4)**	32.4	34.0	33.2^y^				
**Post-inoculation (day 7)**	36.1	39.4	37.8^x^				
**Serum interleukin-6, pg/mL**							
**Pre-inoculation (day** −**1)**	51.3	84.1	67.7^y^	7.99	0.020	<0.001	0.883
**Post-inoculation (day 4)**	98.0	122	110^x^				
**Post-inoculation (day 7)**	70.9	97.6	84.3^y^				
**Serum TNF-α, ρg/mL**							
**Pre-inoculation (day** −**1)**	298	296	297^y^	8.06	0.014	0.004	0.007
**Post-inoculation (day 4)**	305	388	347^x^				
**Post-inoculation (day 7)**	328	335	331^x^				
**Serum diamine oxidase, mg/mL**							
**Pre-inoculation (day** −**1)**	23.8	27.4	25.6^y^	0.63	0.043	<0.001	0.264
**Post-inoculation (day 4)**	35.4	35.4	35.4^x^				
**Post-inoculation (day 7)**	25.1	27.2	26.2^y^				
**Plasma haptoglobin, ng/mL**							
**Pre-inoculation (day** −**1)**	26.2	33.3	29.7^y^	0.93	0.001	<0.001	0.350
**Post-inoculation (day 4)**	37.3	39.6	38.5^x^				
**Post-inoculation (day 7)**	28.5	33.3	30.9^y^				
**Plasma d-Lactate, nmol/mL**							
**Pre-inoculation (day** −**1)**	14.8	16.2	15.5^y^	1.13	0.603	0.034	0.215
**Post-inoculation (day 4)**	17.0	18.9	17.9^xy^				
**Post-inoculation (day 7)**	19.3	17.4	18.3^x^				
**Plasma alkaline phosphatase, IU/L**							
**Pre-inoculation (day 6)**	65.4	63.4	64.4	4.65	0.480	0.330	0.310
**Post-inoculation (day 4)**	73.2	79.5	76.4				
**Post-inoculation (day 7)**	81.3	63.0	72.2				
**Plasma superoxide dismutase, U/mL**							
**Pre-inoculation (day** −**1)**	0.23	0.21	0.22^x^	0.01	0.420	0.001	0.654
**Post-inoculation (day 4)**	0.23	0.2	0.21^x^				
**Post-inoculation (day 7)**	0.12	0.13	0.13^y^				
**Plasma malondialdehyde, nmol/mL**							
**Pre-inoculation (day** −**1)**	0.56	0.58	0.57	0.03	0.367	<0.0001	0.775
**Post-inoculation (day 4)**	1.06	1.14	1.10				
**Post-inoculation (day 7)**	0.67	0.68	0.67				
**Reduced glutathione (GSH), µM**							
**Pre-inoculation (day** −**1)**	3.66	3.07	3.36^x^	0.08	0.001	<0.001	0.331
**Post-inoculation (day 4)**	2.97	2.48	2.73^y^				
**Post-inoculation (day 7)**	3.30	3.14	3.22^x^				
**Oxidized glutathione (GSSG), µM**							
**Pre-inoculation (day** −**1)**	1.16	1.00	1.08	0.04	0.613	0.175	0.050
**Post-inoculation (day 4)**	1.12	1.27	1.20				
**Post-inoculation (day 7)**	1.10	1.18	1.14				
**GSH:GSSG**							
**Pre-inoculation (day** −**1)**	3.28	3.18	3.22^x^	0.13	0.017	0.001	0.396
**Post-inoculation (day 4)**	2.66	1.98	2.32^y^				
**Post-inoculation (day 7)**	3.29	2.66	2.97^x^				
**Plasma urea nitrogen, nmol/µL**							
**Pre-inoculation (day** −**1)**	213	147	180	14.4	0.286	0.442	0.036
**Post-inoculation (day 4)**	184	240	212				
**Post-inoculation (day 7)**	218	161	189				

IDP, indigestible dietary protein; TNF-α, tumor necrosis factor-alpha.

1 Experimental diets with low IDP (LIDP) and high IDP (HIDP). Values are least square means; n = 8 pigs/treatment.

x,y,z In a column, means assigned different lowercase superscript letters are significantly different (*P *< 0.05).

### Ammonia-nitrogen concentrations in intestinal digesta

**Figure 5.**
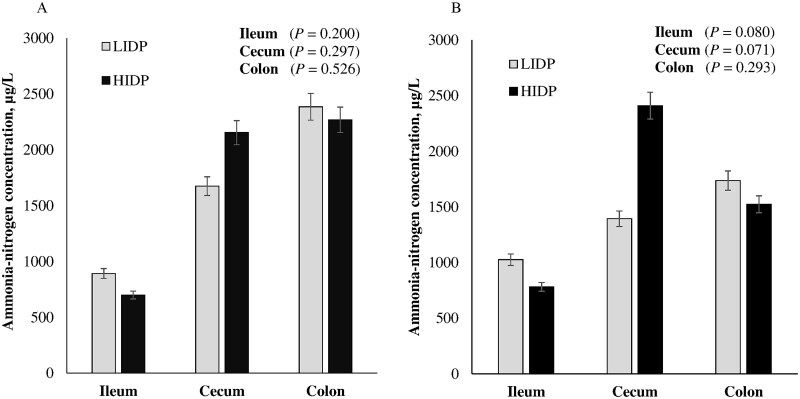
Ammonia-nitrogen concentration in ileal, cecal, and colonic digesta of piglets fed low (LIDP) or high indigestible dietary protein (HIDP) content and inoculated with enterotoxigenic *E. coli* (A) or *Salmonella* Typhimurium (B). Values are least square means ± standard error of mean; n = 8 pigs/treatment.

Ammonia-nitrogen concentration in ileal, cecal, and colonic digesta in pigs challenged with ETEC or ST are shown in Figure 5A and B, respectively. There was no IDP effect on ileal, cecal, and colonic NH_3_-N in ETEC infected-pigs (*P *> 0.10). In ST-pigs, ileal and cecal NH_3_-N tended to be higher and lower in ileal and cecal digesta of LIDP-pigs, respectively, compared to HIDP (*P *< 0.10); however, colonic NH_3_-N was not affected (*P *> 0.10).

### Fecal and digesta myeloperoxidase activity

**Figure 6.**
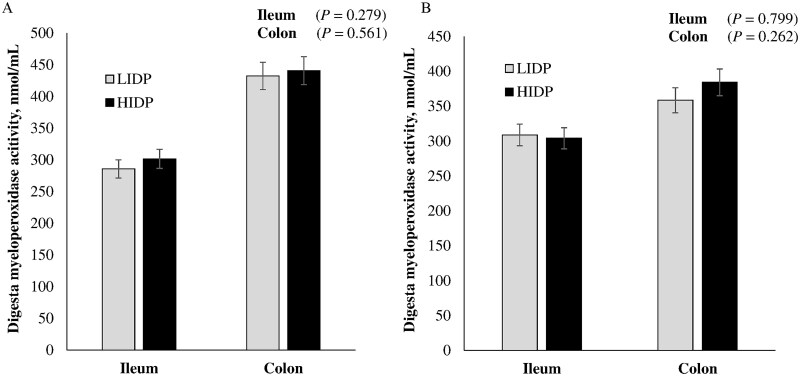
Ileal and colonic myeloperoxidase activity in piglets fed low (LIDP) or high indigestible dietary protein (HIDP) content and inoculated with enterotoxigenic *E. coli* (A) or *Salmonella* Typhimurium (B). Values are least square means ± standard error of the mean; n = 8 pigs/treatment.

**Figure 7.**
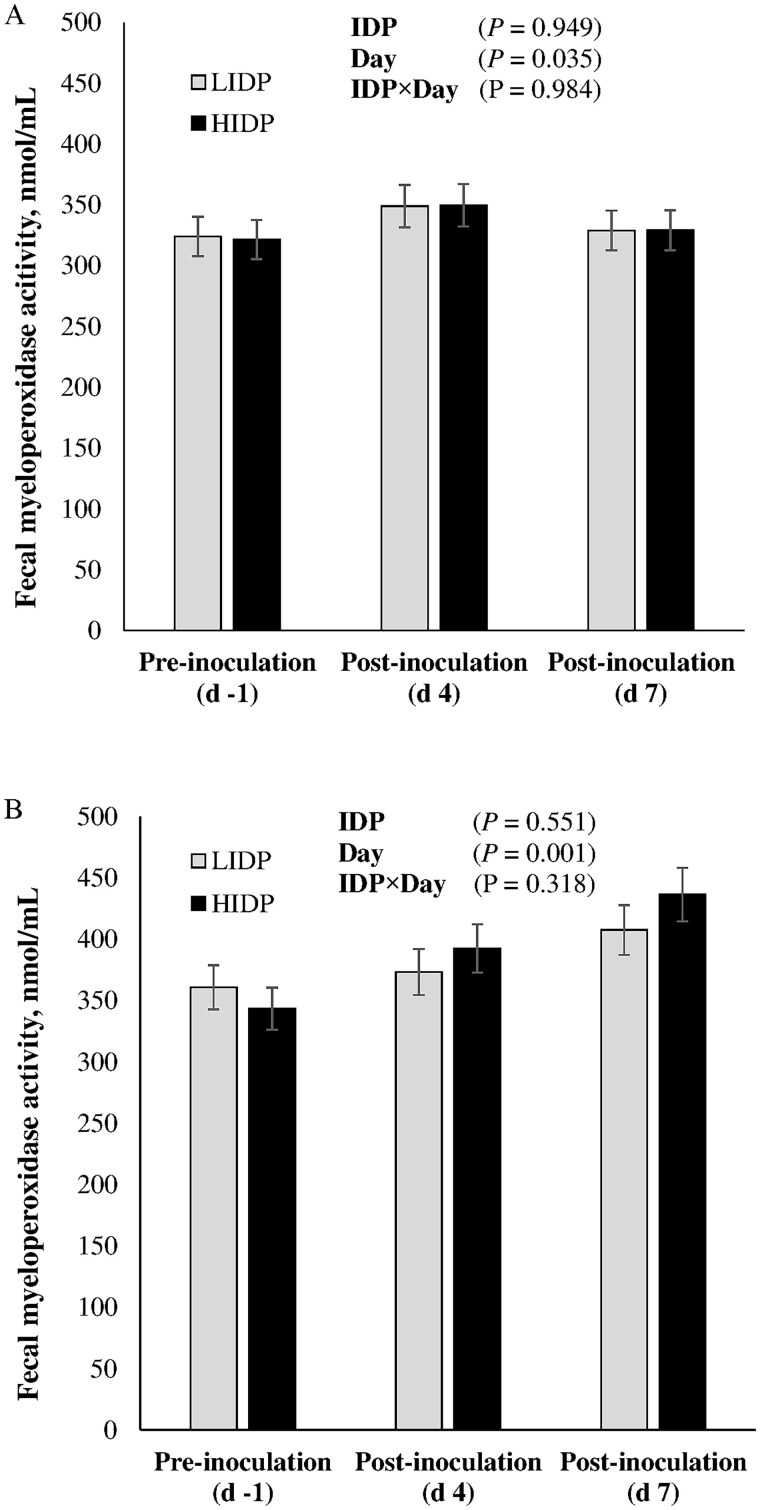
Fecal myeloperoxidase activity in piglets fed low (LIDP) or high indigestible dietary protein (HIDP) content and inoculated with enterotoxigenic *E. coli* (A) or *Salmonella* Typhimurium (B). Values are least square means ± standard error of the mean; n = 8 pigs/treatment.

Pre- and post-inoculation digesta and fecal MPO are shown in Figures 6A and B and 7A and B, respectively. Inoculation with either ETEC or ST did not influence MPO in ileal or colonic digesta (*P *> 0.10). Regardless of pathogen type, there was no IDP effect on fecal MPO (*P *> 0.10); however, a significant increase was observed on day 4 and 7 post-inoculation with ETEC or ST, respectively.

## Discussion

The objective of the present study was to determine the effect of HIDP on growth performance, immune status, and gut health of newly weaned pigs when exposed to either ETEC or ST. The experimental diets were formulated to contain similar crude protein and net energy contents to meet the nutritional requirement of pigs, but differing IDP levels. In the current study, adjustments were made to ingredient content in order to achieve the desired IDP content in our treatment diets, specifically partially replacing soy protein concentrate and corn with corn DDGS and canola meal to achieve the HIDP diet. While adjusting IDP by replacing animal with plant-based sources would have resulted in a greater difference in IDP across our treatments (de Oliveira et al., 2025), animal-based protein sources were avoided in order to reduce the confound and potential positive effects of animal-based sources (Rodrigues et al., 2022). Moreover, the choice to use typical feed ingredients, rather than purified diets, resulted in unavoidable differences in nutrient content (e.g., increased fiber with increasing IDP). This is common in nutrition studies, especially those studying changes in IDP (Wellington et al., 2020). As fiber is typically suggested as a method to reduce negative effects of high HIDP, we do not feel that this would not have had a significant effect on the interpretation of effects of IDP in diets and, at most, would have reduced the effects of the HIDP treatment. However, results should be interpreted with this in mind.

Previously, we reported that HIDP increased diarrhea severity while reducing growth response and oxidative status of weaning pigs (Erinle et al., 2025a). However, these results were observed in healthy pigs and the interaction of IDP and enteric pathogenic has not been determined. Understanding the implications of IDP content on the growth and gut health of nursery pigs would be of great importance to the swine industry, particularly when formulating diets to improve weaning outcomes and gut health robustness under disease challenge. To our knowledge, this is the first study to report the effect of IDP on growth performance, immune, and gut health of piglets challenged with ETEC or ST.

### Effects of IDP on rectal temperature, fecal consistency score, and NH_3_-N concentrations in weaning pigs challenged with ETEC or ST inoculation

Newly weaned pigs are highly susceptible to enteric pathogenic infection due to their immature mucosal immunity. At the acute stage, an increase in core body temperature (i.e., fever) can be among the first clinical signs of pathogenic infection in animals, including pigs (Martínez‐Avilés et al., 2017). Enterotoxigenic *E. coli* infections are variably associated with increased rectal temperature post-infection (Yi et al., 2005; Rhouma et al., 2017b; Choi et al., 2020; Jin et al., 2024). Studies involving ETEC challenge in pigs demonstrated a maximum increase in rectal temperature, usually between 6 and 12 h post-infection (Yi et al., 2005; Choi et al., 2021) or 24 h post-infection (Jin et al., 2024). In our study, inoculation with ETEC at 1.36 × 10^6^ CFU·mL^−1^ resulted in a slight increase in rectal temperature at 8 h post-inoculation; however, the mean temperature remained within the normal range for nursery pigs. In contrast to our findings, Choi et al. (2021) reported a significant increase in rectal temperature in piglets challenged with 1 × 10^7^ CFU·mL^−1^ ETEC. However, at a higher concentration, pigs inoculated with ETEC of 10^9^ CFU·mL^−1^ did not have increased rectal temperatures (Rhouma et al., 2017b). Despite the high inoculum concentration and absence of fever, Rhouma et al. (2017b) reported that the ETEC encoding both enterotoxin and F4 genes were recovered from the feces of the pigs. This suggests that the absence of fever does not necessarily imply an absence of ETEC infection. In addition to fever, FCS is used as a prognostic evaluation of diarrhea severity and prevalence, with a higher FCS associated with severe diarrhea (Renaud et al., 2020; Erinle et al., 2025b). While we did not detect a clear sign of fever, ETEC inoculation increased FCS, in which normal to mild diarrhea was observed throughout the post-inoculation period. Similar to the reports from several studies, ETEC infection induces diarrhea in piglets (Girard et al., 2018; Choi et al., 2020; Xu et al., 2023). However, we did not observe IDP effect on FCS in ETEC-infected pigs. In contrast to the present findings, we have previously reported a higher FCS in healthy pigs fed HIDP during day 5 to 7 post-weaning (Erinle et al., 2025a). While the physiological reason for the contrasting effect of IDP on FCS between the present and previous studies is unclear, it could be due to the difference in the housing pattern employed across both studies (individual- vs. group-housing). Given the insignificant effect of IDP on FCS in ETEC-pigs, it is apparent that the diarrhea severity we observed was due to ETEC infection.

Contrastingly, we observed a clear elevation of rectal temperature and poor FCS in pigs inoculated with ST at 1.14 × 10^10^ CFU·mL^−1^. In agreement with our findings, Rodrigues et al. (2021) reported an increased rectal temperature and deterioration of FCS in pigs challenged with 1.33 × 10^9^ CFU·mL^−1^. After oral inoculation, ST has been reported to be mostly localized in the ileum and the large intestine of piglets and cause infection (Bescucci et al., 2020; Radlinski et al., 2024). Upon colonization of the GIT, ST produce several virulence factors, including the type III secretion system used to secrete proteins into host cells (Collazo and Galán, 1997). These virulence factors trigger fever, diarrhea in young pigs (Tanaka et al., 2010; Yin et al., 2014) and inflammation (Bescucci et al., 2020). Unlike during pre-inoculation periods, we observed poor FCS in HIDP-fed pigs following ST inoculation compared to those fed LIDP. Although pathogen-specific, the poor FCS partly supports our hypothesis that HIDP diet would increase diarrhea severity under enteric pathogenic infection. Different IDP levels might impact several factors important for infection including the microbiota, production and availability of secondary metabolites, as well as amino acids which can all impact bacterial virulence (Bhagwat et al., 2025).

A potential explanation for the difference observed in impact of HIDP in nursery pigs infected with ETEC and ST might be linked to the contrasting site of colonization of the two pathogens in the GIT and the relevance of the site in microbial protein fermentation. Enterotoxigenic *E. coli* has been shown to colonize the upper GIT (Choi et al., 2020), while ST colonizes the distal GIT as their primary site of involvement (Darwin and Miller, 1999; Bellido-Carreras et al., 2019). On top of that, ST interferes with the absorption of amino acids in the small intestine, thereby promoting their entrance into the large intestine, where they could be fermented (Radlinski et al., 2024). Since ST colonizes the distal GIT, it is plausible that pigs fed HIDP may experience more pronounced protein fermentation, which could, in turn, contribute to the poor FCS observed in ST-challenged pigs compared with ETEC-challenged pigs.

In the present study, there was no IDP effect on NH_3_-N concentrations in ileal, cecal, and colonic digesta in ETEC-pigs. Meanwhile, in pigs challenged with ST, HIDP tended to reduce ileal and increase cecal NH_3_-N concentrations compared to LIDP-fed pigs. The cecum appeared to be the main site of microbial fermentation of IDP, as we have previously reported that IDP influenced more protein fermentation metabolites in the cecum compared to colon (Erinle et al., 2025a). High concentration of NH_3_-N is strongly associated with the incidence of PWD (Jha and Berrocoso, 2016). The trend toward higher NH_3_-N in cecum of HIDP-fed pigs under ST challenge could help explain, at least in part, the poor FCS noted, suggesting a possible link between increased protein fermentation and diarrhea severity. Therefore, future studies should focus on deciphering pathogen-specific impacts of varying IDP.

### Effects of IDP content on growth response of weaning pigs challenged ETEC or ST inoculation

Reducing DP from 22% to 16% was reported to reduce ADG, G: F, and final BW (Fang et al., 2019; Limbach et al., 2021). However, other studies also demonstrated that increasing DP from 16% to 20% did not affected growth performance (Rodrigues et al., 2021), while 17.6% to 24% DP diet improve ADG in weaning pigs (Opapeju et al., 2008, 2009; Diether et al., 2024). Thus, varying dietary DP levels have contradicting effects on growth performance of weaning pigs, and this could be due the varying extent of digestibility of composite protein ingredients in the diet. Since the diets in the present study were formulated to contain similar crude protein and net energy contents to meet the nutritional requirement of pigs, it should, in principle, not impact growth performance. Prior to inoculation with ETEC or ST (day −7 to 0), we did not observe IDP effect on growth performance of pigs. This is in line with our previous findings (Erinle et al., 2025a), where we saw no difference in growth performance of unchallenged pigs fed LIDP or HIDP during the first 2 w post-weaning. However, at day 7 post-inoculation in the current study, we observed a significant reduction in ADFI, ADG, and final BW in ST-pigs fed HIDP compared to LIDP. Meanwhile, this similar effect was not observed in ETEC-inoculated pigs. In general, inoculation of ETEC (Boeckman et al., 2022; Xu et al., 2023) or ST (Rodrigues et al., 2021; Won et al., 2021) has consistently been shown to reduce growth performance response of weaning pigs. The negative effect of IDP on growth performance has also been reported in other immune-stimulated animal species. For example, HIDP was shown to reduce body weight gain and ileal nitrogen digestibility in broiler chicken, which was further reduced when challenged with *Eimeria tenella* compared with the non-challenged groups (Sung and Adeola, 2025). Whether nutritional or pathogenic, diarrheal disease is often symptomized by dehydration, reduced feed intake, and consequently accounts for a 20% reduction in body weight in children (Black et al., 1984). Thus, the negative impact on growth performance of ST-pigs might be related to the diarrhea severity (i.e., FCS) and gut health impairment caused by the combined effect of ST challenge and IDP content, which was partially absent in ETEC-pigs.

### Effects of IDP content on intestinal inflammation, oxidative status, and immune system stimulation biomarkers in weaning pigs challenged ETEC or ST inoculation

The presence of undigested protein in the large intestine poses varied impacts on gut health, including but not limited to its interference with oxidative status, inflammation of epithelial cells that could cause chloride ion secretion, and PWD (Pieper et al., 2016; Blanch 2018; Diether and Willing, 2019). In the present study, IDP did not influence MPO activity in the ileal, colonic, and fecal content, however, intestinal inflammation was induced, as evidenced by an increase in fecal MPO activity following ETEC or ST inoculation. Myeloperoxidase is a lysosomal component of neutrophils and is often released when degranulated to kill intruding organisms. However, their presence in the feces is sought as a non-invasive biomarker of intestinal inflammation caused by bacterial antigens (Hansberry et al., 2017). Upregulation of MPO was demonstrated in weaned pigs inoculated with either ETEC or ST (Rodrigues et al., 2021; Li et al., 2024).

Following the breach of mucosal barrier, the translocation pathogenic bacteria invoke an intestinal acute inflammatory response capable of stimulating the production of cytokines at the site of inflammation and travel through the bloodstream. In the present study, we observed stimulation of immune response and antioxidant status as indicated by altering level of acute-phase protein and antioxidant enzymes in the blood pigs inoculated with ETEC or ST. In ETEC, HIDP-fed pigs had an increased serum IL-6, reduced PUN, and a tendency to increase and reduce plasma haptoglobin and GSH. Plasma urea nitrogen is used in the assessment of DP and amino acids utilization, with reduced PUN indicating high utilization of amino acids (Marín-García et al., 2022). This implies that pigs fed HIDP diets were more efficient in utilizing DP or amino acids under ETEC challenge compared to LIDP; however, the low PUN did not translate to improved growth performance. Furthermore, high DP diets (usually > 20%) have been shown to cause an over-expression of mucosal inflammatory biomarkers, including IL-6, TNF-α, IL-8, IL-1β, IFN-γ, and NF-kB/MAPK signaling pathway (Pieper et al., 2012; Gao et al., 2020; Liu et al., 2022; Xia et al., 2022) and increase serum urea nitrogen (Zhao et al., 2021). In contrast to the pattern observed in ETEC, HIDP had elevated serum ALB, IL-6, TNF-α, DAO, plasma haptoglobin, and GSH, with a depressed level of GSH:GSSG in ST-pigs. Elevated serum levels of ALB and pro-inflammatory cytokines, including IL-6, IL-1β, and TNF-α, have been implicated in GIT inflammation and infections (Limbach et al., 2021). Diamine oxidase is produced in epithelial enterocytes to catalyze the oxidative deamination of diamines and polyamines and has been sought as an indication of compromised gut integrity in weaned pigs (Tossou et al., 2016). Thus, the higher DAO levels among the ST-pigs is an indication that HIDP diet could increase the amount of DP and amino acids reaching the hindgut. Furthermore, inflammatory responses in pigs have been associated with increased haptoglobin levels, a positive acute phase protein (Limbach et al., 2021). This indicates an escalation of immune response HIDP-fed pigs that were infected with ST compared to ETEC.

In addition to immune response, enzymatic antioxidants, including GSH and SOD, continuously help to protect the GIT against acute or chronic oxidative stress. Reduced glutathione neutralizes free radicals, particularly reactive oxygen species, oxidizing it to GSSG. Consequently, a reduction in GSH:GSSH ratio suggests a depletion of enzymatic antioxidants in the body, which could further increase the susceptibility of pigs to oxidative stress (Jin et al., 2024). This is in agreement with the observed reduction in growth performance and increased FCS in ST-pigs, an effect which appears to be milder in ETEC-infected pigs. Taken together, HIDP diet promoted pathogen population in the ileum and cecum, aided their extracellular translocation, provoked immune responses, and distorted antioxidant balance in ST-infected pigs.

### Effects of IDP content on pathogenic quantification in digesta and their translocation into MLN, spleen, and liver of weaning pigs challenged ETEC or ST inoculation

Mucosal surfaces, especially in the GIT, provide the entry point for over 90% of pathogens affecting humans and animals and, as a result, are designated as the first line of defense against infection (Miquel-Clopés et al., 2019; Song et al., 2024). An increase in opportunistic pathogens would be expected in the distal GIT as the main site of DP fermentation (Ball and Aherne, 1987; Htoo et al., 2007). A significant increase in the translocation of pathogens from the GIT to MLN and later to other regional tissues has been implicated in many immuno-inflammatory disease conditions (Paino et al., 2019; Jin et al., 2022), causing an alteration in the structural integrity of mucosal barrier. Once in the MLN, ST could evade cytotoxic immune response in weaning pigs by reducing only major histocompatibility complex II in infected cells, thereby limiting antigen presentation to CD4^+^ T cells (Martins et al., 2013). However, this is not the case in piglets infected with ETEC as the host has been shown to clear the pathogen usually at day 7 post-inoculation via the secretion of IL-17 as an innate immune response (Verdonck et al., 2002; Luo et al., 2015). In the present study, we detected the presence of ETEC or ST in the ileal, cecal, colonic, MLN, liver, and spleen of pigs on day 7 post-inoculation. In agreement with our study, Rodrigues et al. (2021) and Wellington et al. (2019) reported the presence of ST in ileum, cecum, colon, MLN, or cecum of pigs on the day 7 post-inoculation with ST, while Choi et al. (2020) detected ETEC gene in colonic digesta on day 5 of pigs. Unlike in ETEC-pigs, LIDP-fed pigs had reduced ST quantification in the cecal digesta and a tendency of reduction in ileal digesta compared to HIDP pigs, which resulted in a lower ST translocation to MLN. Interestingly, feeding a less digestible protein diets using plant sources was reported to increase the susceptibility of weaning pigs to ST compared a more digestible protein diets using animal-based protein sources (Rodrigues et al., 2022). It is not clear whether the increase in ST in the cecal and ileal content was due to HIDP, however, ST often show a phenotypic diversity and motility toward the direction of nutrients and thrive where the concentration of nutrients is plentiful (Koirala et al., 2014). This suggests that the presence of undigested protein matrix from HIDP could provide a thriving condition for ST. Taken together, an increase in IDP fraction contributes to ST migration into the ileum and cecum of ST-infected pigs and increases their translocation to the MLN, most likely through reduced barrier function as a result of production of harmful metabolites through fermentation, thereby promoting diarrhea severity and reducing growth performance in nursery pigs.

## Conclusions

In summary, our results showed that the impact of IDP on newly weaned pigs is dependent on the enteric pathogen type. Under ETEC F4 challenge, HIDP had minimal effect on clinical, physiological, and performance outcomes, suggesting milder exacerbated acute response. In contrast, when pigs were challenged with ST, HIDP intensified diarrhea severity, impaired growth, and promoted pathogen colonization and translocation, accompanied by stronger inflammatory and redox responses. These patterns indicated that the distal GIT, where both IDP fermentation and ST activity were most pronounced, may be a critical site driving the differential outcomes. Overall, at a similar DP level, feeding newly weaned pigs with LIDP diets may be a worthwhile dietary strategy to improve weaning experience caused by exposure to enteric pathogen challenge, particularly ST infection.

## Data Availability

Data available upon reasonable request to the corresponding author.

## Abbreviations:

AA: amino acid
ADFI: average daily feed intake
ADG: average daily gain
ALB: albumin
ALP: alkaline phosphatase
BPW: buffered peptone water
BW: body weight
DP: crude protein
DAO: diamine oxidase
DM: dry matter
DP: dietary protein
ETEC: enterotoxigenic *Escherichia coli*
G:F: gain:feed
GIT: ­gastrointestinal tract
GSH: reduced glutathione
GSSG: oxidized glutathione
HIDP: high indigestible dietary protein
IDP: indigestible dietary protein
IL-6: interleukin-6
LIDP: low indigestible dietary protein
MDA: malondialdehyde
ME: metabolizable energy
MLN: mesenteric lymph nodes
MPO: myeloperoxidase
NE: net energy
NH_3_-N: ammonia-nitrogen
PUN: plasma urea nitrogen
PWD: post-weaning diarrhea
SID: standardized ileal digestible
SOD: superoxide dismutase
ST: *Salmonella enterica* subspecies *enterica* serovar Typhimurium
TNF-α: tumor necrosis factor alpha

## References

[skaf451-B2085184] Ball R. O and Aherne F. X. 1987. Influence of dietary nutrient density, level of feed intake and weaning age on young pigs. II. Apparent nutrient digestibility and incidence and severity of diarrhea. Can. J. Anim. Sci. 67(4):1105–1115. 10.4141/cjas87-116

[skaf451-B2] Bao H. , Wang S., Zhao J. H., Liu S. L. 2020. Salmonella secretion systems: differential roles in pathogen-host interactions. Microbiol. Res. 241:126591. http://doi:10.1016/j.micres.2020.126591 32932132 10.1016/j.micres.2020.126591

[skaf451-B4] Bellido-Carreras N. , Argüello H., Zaldívar-López S., Jiménez-Marín Á., Martins R. P., Arce C., Morera L., Carvajal A., Garrido J. J. 2019. *Salmonella* Typhimurium infection along the porcine gastrointestinal tract and associated lymphoid tissues. Vet. Pathol. 56(5):681–690. http://doi:10.1177/0300985819843682 31106677 10.1177/0300985819843682

[skaf451-B5] Bescucci D. M. , Moote P. E., Ortega Polo R., Uwiera R. R., Inglis G. D. 2020. *Salmonella* enterica serovar Typhimurium temporally modulates the enteric microbiota and host responses to overcome colonization resistance in swine. Appl. Environ. Microbiol. 86(21):e01569-20. http://doi:10.1128/AEM.01569-20 32859592 10.1128/AEM.01569-20 PMC7580545

[skaf451-B6] Bhagwat A. , Haldar T., Kanojiya P., Saroj S. D. 2025. Bacterial metabolism in the host and its association with virulence. Virulence. 16(1):2459336. http://doi:10.1080/21505594.2025.2459336 39890585 10.1080/21505594.2025.2459336 PMC11792850

[skaf451-B7] Black R. E. , Brown K. H., Becker S. 1984. Effects of diarrhea associated with specific enteropathogens on the growth of children in rural Bangladesh. Pediatr. 73(6):799–805. http://doi:10.1542/peds.73.6.799 6374599

[skaf451-B8] Blanch A. 2018. Undigested feed protein affects pigs’ health. Available from https://www.pig333.com/articles/undigested-feed-protein-affects-pigs-health_13249

[skaf451-B5642770] Boeckman J. X. , Sprayberry S., Korn A. M., Suchodolski J. S., Paulk C., Genovese K., Rech R. R., Giaretta P. R., Blick A. K., Callaway T. et al. 2022. Effect of chronic and acute enterotoxigenic E. coli challenge on growth performance, intestinal inflammation, microbiome, and metabolome of weaned piglets. Sci. Rep. 12(1):5024. 10.1038/s41598-022-08446-z PMC8943154 35323827

[skaf451-B10] Canadian Council on Animal Care. 2009. The care and use of farm animals in research, teaching and testing. Ottawa: CCAC; p 12–15.

[skaf451-B11] Choi J. , Wang L., Liu S., Lu P., Zhao X., Liu H., Lahaye L., Santin E., Liu S., Nyachoti M. et al. 2020. Effects of a microencapsulated formula of organic acids and essential oils on nutrient absorption, immunity, gut barrier function, and abundance of enterotoxigenic *Escherichia coli* F4 in weaned piglets challenged with *E. coli* F4. J. Anim. Sci. 98(9): skaa259. http://doi:10.1093/jas/skaa259 32780110 10.1093/jas/skaa259 PMC7526869

[skaf451-B12] Collazo C. M. , Galán J. E. 1997. The invasion‐associated type III system of *Salmonella* typhimurium directs the translocation of sip proteins into the host cell. Mol. Microbiol. 24(4):747–756. http://doi:10.1046/j.1365-2958.1997.3781740.x 9194702 10.1046/j.1365-2958.1997.3781740.x

[skaf451-B13] Darwin K. H. , Miller V. L. 1999. Molecular basis of the interaction of *Salmonella* with the intestinal mucosa. Clin. Microbiol. Rev. 12(3):405–428. http://doi:10.1128/cmr.12.3.405 10398673 10.1128/cmr.12.3.405 PMC100246

[skaf451-B15] Diether N. E. , Kommadath A., Fouhse J. M., Zijlstra R. T., Stothard P., Willing B. P. 2024. Increased dietary protein rather than fiber supports key metabolic and intestinal tissue functions in pigs, without increasing post-weaning diarrhea. Am. J. Physiol. Gastrointest. Liver Physiol. 327(6):G818–G831. http://doi:10.1152/ajpgi.00146.2024 39406387 10.1152/ajpgi.00146.2024

[skaf451-B16] Diether N. E. , Willing B. P. 2019. Microbial fermentation of dietary protein: an important factor in diet–microbe–host interaction. Microorganisms. 7(1):19. http://doi:10.3390/microorganisms7010019 30642098 10.3390/microorganisms7010019 PMC6352118

[skaf451-B17] de Oliveira M. J. K. , Babatunde O. O., Rodrigues L. A., Erinle T. J., Htoo J. K., Mendoza S. M., Columbus D. A. 2025. Development of an indigestible dietary protein index to investigate the effects of dietary protein content in post-weaning pigs. J. Anim. Sci. 103:skae374. http://doi:10.1093/jas/skae374 39657758 10.1093/jas/skae374 PMC11705088

[skaf451-B18] Drumo R. , Pesciaroli M., Ruggeri J., Tarantino M., Chirullo B., Pistoia C., Petrucci P., Martinelli N., Moscati L., Manuali E. et al. 2015. *Salmonella enterica* serovar typhimurium exploits inflammation to modify swine intestinal microbiota. Front. Cell. Infect. Microbiol. 5:106. http://doi:10.3389/fcimb.2015.00106 26835435 10.3389/fcimb.2015.00106 PMC4722131

[skaf451-B19] Erinle T. J. , de Oliveira M.J.K, Htoo J. K., Mendoza S. M., Columbus D. A. 2025a. Effects of indigestible dietary protein on growth performance and health status of weaned pigs. J. Anim. Sci. J. Anim. Sci. 103:skaf372. http://doi:10.1093/jas/skaf372 41138160 10.1093/jas/skaf372 PMC12620005

[skaf451-B20] Erinle T. J. , Babatunde O. O., Htoo J. K., Mendoza S. M., Columbus D. A. 2025b. Dietary fibre fractions supplementation modulates pro-inflammatory cytokines, hindgut fermentation metabolites, and fecal consistency score in nursery pigs. Can. J. Anim. Sci. JUST-IN. 105:1. http://doi:10.1139/cjas-2024-0123

[skaf451-B21] Evonik. 2016. AMINODat^®^5.0 Platinum version. Hanau-Wolfgang, Germany: Evonik Nutrition and Care GmbH.

[skaf451-B22] Fang L. H. , Jin Y. H., Do S. H., Hong J. S., Kim B. O., Han T. H., Kim Y. Y. 2019. Effects of dietary energy and crude protein levels on growth performance, blood profiles, and nutrient digestibility in weaning pigs. Asian-Australas. J. Anim. Sci. 32(4):556–563. http://doi:10.5713/ajas.18.0294 30145868 10.5713/ajas.18.0294 PMC6409451

[skaf451-B23] Fleckenstein J. M. , Hardwidge P. R., Munson G. P., Rasko D. A., Sommerfelt H., Steinsland H. 2010. Molecular mechanisms of enterotoxigenic *Escherichia coli* infection. Microbes Infect. 12(2):89–98. http://doi:10.1016/j.micinf.2009.10.002 19883790 10.1016/j.micinf.2009.10.002 PMC10647112

[skaf451-B24] Gao J. , Yin J., Xu K., Han H., Liu Z., Wang C., Li T., Yin Y. 2020. Protein level and infantile diarrhea in a post-weaning piglet model. Mediators Inflamm. 2020:1937387. http://doi:10.1155/2020/1937387 32565721 10.1155/2020/1937387 PMC7281817

[skaf451-B25] García V. , Gambino M., Pedersen K., Haugegaard S., Olsen J. E., Herrero-Fresno A. 2020. F4- and F18-positive enterotoxigenic Escherichia coli isolates from diarrhea of postweaning pigs: genomic characterization. Appl. Environ. Microbiol. 86(23):e01913-20. http://doi:10.1128/AEM.01913-20 10.1128/AEM.01913-20 PMC7657637 32948526

[skaf451-B26] Girard M. , Thanner S., Pradervand N., Hu D., Ollagnier C., Bee G. 2018. Hydrolysable chestnut tannins for reduction of post-weaning diarrhea: Efficacy on an experimental ETEC model. PLoS One. 13(5):e0197878. 29799865 10.1371/journal.pone.0197878 PMC5969761

[skaf451-B27] Hansberry D. R. , Shah K., Agarwal P., Agarwal N. 2017. Fecal myeloperoxidase as a biomarker for inflammatory bowel disease. Cureus. 9(1):e1004. http://doi:10.7759/cureus.1004 28286723 10.7759/cureus.1004 PMC5332167

[skaf451-B28] Htoo J. K. , Araiza B. A., Sauer W. C., Rademacher M., Zhang Y., Cervantes M., Zijlstra R. T. 2007. Effect of dietary protein content on ileal amino acid digestibility, growth performance, and formation of microbial metabolites in ileal and cecal digesta of early-weaned pigs. J. Anim. Sci. 85(12):3303–3312. http://doi:10.2527/jas.2007-0105 17785591 10.2527/jas.2007-0105

[skaf451-B29] Jayaraman B. , Regassa A., Htoo J. K., Nyachoti C. M. 2017. Effects of dietary standardized ileal digestible tryptophan: lysine ratio on performance, plasma urea nitrogen, ileal histomorphology and immune responses in weaned pigs challenged with *Escherichia coli* K88. Livest. Sci. 203:114–119. http://doi:10.1016/J.LIVSCI.2017.07.014

[skaf451-B30] Jin S. , Wetzel D., Schirmer M. 2022. Deciphering mechanisms and implications of bacterial translocation in human health and disease. Curr. Opin. Microbiol. 67:102147. 10.1016/j.mib.2022.102147 35461008

[skaf451-B31] Jin S. , Xu H., Yang C., Karmin O. 2024. Regulation of oxidative stress in the intestine of piglets after enterotoxigenic *Escherichia coli* (ETEC) infection. Biochim. Biophys. Acta Mol. Cell Res. 1871(5):119711. http://doi:10.1016/j.bbamcr.2024.119711 38574824 10.1016/j.bbamcr.2024.119711

[skaf451-B32] Jensen G. M. , Frydendahl K., Svendsen O., Jørgensen C. B., Cirera S., Fredholm M., Nielsen J. P., Møller K. 2006. Experimental infection with *Escherichia coli* O149: F4ac in weaned piglets. Vet. Microbiol. 115(1–3):243–249. http://doi:10.1016/j.vetmic.2006.01.002 16466864 10.1016/j.vetmic.2006.01.002

[skaf451-B27617567] Jha R and J. F. Berrocoso. 2016. Dietary fiber and protein fermentation in the intestine of swine and their interactive effects on gut health and on the environment: A review. Anim. Feed Sci. Technol. 212:18–26. 10.1016/j.anifeedsci.2015.12.002

[skaf451-B33] Jørgensen M. G. , van Raaphorst R., Veening J. W. 2013. Noise and stochasticity in gene expression: a pathogenic fate determinant. Methods Microbiol. 40:157–175. 10.1016/B978-0-12-417029-2.00006-6

[skaf451-B34] Koirala S. , Mears P., Sim M., Golding I., Chemla Y. R., Aldridge P. D., Rao C. V. 2014. A nutrient-tunable bistable switch controls motility in *Salmonella enterica* serovar typhimurium. mBio. 5(5):e01611–e01614. http://doi:10.1128/mbio.01611-14 25161191 10.1128/mBio.01611-14 PMC4173784

[skaf451-B35] Li L. , Han K., Mao X., Wang L., Cao Y., Li Z., Wu Y., Tan Y., Shi Y., Zhang L. et al. 2024. Oral phages prophylaxis against mixed *Escherichia coli* O157: H7 and *Salmonella* typhimurium infections in weaned piglets. Vet. Microbiol. 288:109923. http://doi:10.1016/j.vetmic.2023.109923 38061277 10.1016/j.vetmic.2023.109923

[skaf451-B36] Li M. , Zhao D., Guo J., Pan T., Niu T., Jiang Y., Shi C., Huang H., Wang N., Zhang D. et al. 2024. Bacillus halotolerans SW207 alleviates enterotoxigenic *Escherichia coli*-induced inflammatory responses in weaned piglets by modulating the intestinal epithelial barrier, the TLR4/MyD88/NF-κB pathway, and intestinal microbiota. Microbiol. Spectr. 12(4):e03988-23. http://doi:10.1128/spectrum.03988-23 38451226 10.1128/spectrum.03988-23 PMC10986599

[skaf451-B37] Li R. , Hou G., Jiang X., Song Z., Fan Z., Hou D. X., He X. 2019. Different dietary protein sources in low protein diets regulate colonic microbiota and barrier function in a piglet model. Food Funct. 10(10):6417–6428. http://doi:10.1039/C9FO01154D 31517363 10.1039/c9fo01154d

[skaf451-B38] Limbach J. R. , Espinosa C. D., Perez-Calvo E., Stein H. H. 2021. Effect of dietary crude protein level on growth performance, blood characteristics, and indicators of intestinal health in weanling pigs. J. Anim. Sci. 99(6):skab166. http://doi:10.1093/jas/skab166 34019637 10.1093/jas/skab166 PMC8202089

[skaf451-B39] Liu Y. , Azad M. A. K., Zhao X., Zhu Q., Kong X. 2022. Dietary crude protein levels alter diarrhea incidence, immunity, and intestinal barrier function of huanjiang mini-pigs during different growth stages. Front. Immunol. 13:908753. http://doi:10.3389/fimmu.2022.908753 35874746 10.3389/fimmu.2022.908753 PMC9301461

[skaf451-B40] Luise D. , Chalvon-Demersay T., Lambert W., Bosi P., Trevisi P. 2021. Meta-analysis to evaluate the impact of the reduction of dietary crude protein on the gut health of post-weaning pigs. Ital. J. Anim. Sci. 20(1):1386–1397. http://doi:10.1080/1828051X.2021.1952911

[skaf451-B41] Luo Y. , Van Nguyen U., de la Fe Rodriguez P. Y., Devriendt B., Cox E. 2015. F4+ ETEC infection and oral immunization with F4 fimbriae elicits an IL-17-dominated immune response. Vet. Res. 46:121–114. http://doi:10.1186/s13567-015-0264-2 26490738 10.1186/s13567-015-0264-2 PMC4618862

[skaf451-B42] Luppi A. 2017. Swine enteric colibacillosis: diagnosis, therapy and antimicrobial resistance. Porc. Health Manag. 3:1–18. http://doi:10.1186/s40813-017-0063-4 10.1186/s40813-017-0063-4 PMC5547460 28794894

[skaf451-B43] Marchetti R. , Faeti V., Gallo M., Pindo M., Bochicchio D., Buttazzoni L., Della Casa G. 2023. Protein content in the diet influences growth and diarrhea in weaning piglets. Animals. (Basel). 13(5):795. http://doi:10.3390/ani13050795 36899653 10.3390/ani13050795 PMC10000050

[skaf451-B44] Marín-García P. J. , Llobat L., López-Lujan M. C., Cambra-López M., Blas E., Pascual J. J. 2022. Urea nitrogen metabolite can contribute to implementing the ideal protein concept in monogastric animals. Animals (Basel). 12(18):2344. http://doi:10.3390/ani12182344 36139206 10.3390/ani12182344 PMC9495106

[skaf451-B45] Martínez‐Avilés M. , Fernández‐Carrión E., López García‐Baones J. M., Sánchez‐Vizcaíno J. M. 2017. Early detection of infection in pigs through an online monitoring system. Transbound. Emerg. Dis. 64(2):364–373. http://doi:10.1111/tbed.12372 25955521 10.1111/tbed.12372

[skaf451-B46] Martins R. P. , Aguilar C., Graham J. E., Carvajal A., Bautista R., Claros M. G., Garrido J. J. 2013. Pyroptosis and adaptive immunity mechanisms are promptly engendered in mesenteric lymph-nodes during pig infections with *Salmonella enterica* serovar typhimurium. Vet. Res. 44(1):120–114. http://doi:10.1186/1297-9716-44-120 24308825 10.1186/1297-9716-44-120 PMC4028780

[skaf451-B48] Miquel-Clopés A. , Bentley E. G., Stewart J. P., Carding S. R. 2019. Mucosal vaccines and technology. Clin. Exp. Immunol. 196(2):205–214. http://doi:10.1111/cei.13285 30963541 10.1111/cei.13285 PMC6468177

[skaf451-B49] Mueller M. , Tainter C. R. 2023. *Escherichia coli* Infection. [Online] Available: https://www.ncbi.nlm.nih.gov/books/NBK564298/33231968

[skaf451-B50] Müller A. J. , Kaiser P., Dittmar K. E., Weber T. C., Haueter S., Endt K., Songhet P., Zellweger C., Kremer M., Fehling H. J. et al. 2012. Salmonella gut invasion involves TTSS-2-dependent epithelial traversal, basolateral exit, and uptake by epithelium-sampling lamina propria phagocytes. Cell Host Microbe. 11(1):19–32. http://doi:10.1016/j.chom.2011.11.013 22264510 10.1016/j.chom.2011.11.013

[skaf451-B51] National Research Council (NRC). 2012. Nutrient requirements of swine. 11th rev. ed. Washington (DC): National Academies Press.

[skaf451-B52] Opapeju F. O. , Krause D. O., Payne R. L., Rademacher M., Nyachoti C. M. 2009. Effect of dietary protein level on growth performance, indicators of enteric health, and gastrointestinal microbial ecology of weaned pigs induced with post-weaning colibacillosis. J. Anim. Sci. 87(8):2635–2643. http://doi:10.2527/jas.2008-1310 19395520 10.2527/jas.2008-1310

[skaf451-B53] Opapeju F. O. , Rademacher M., Blank G., Nyachoti C. M. 2008. Effect of low-protein amino acid-supplemented diets on the growth performance, gut morphology, organ weights and digesta characteristics of weaned pigs. Animals. 2(10):1457–1464. http://doi:10.1017/S175173110800270X 10.1017/S175173110800270X 22443903

[skaf451-B55] Pearce S.C. , Nisley M.J., Kerr B.J., Sparks C., Gabler N.K. 2024. Effects of dietary protein level on intestinal function and inflammation in nursery pigs. J. Anim. Sci. 102: skae077. http://doi:10.1093/jas/skae077 38504643 10.1093/jas/skae077 PMC11015048

[skaf451-B57] Pieper R. , Tudela C. V., Taciak M., Bindelle J., Pérez J. F., Zentek J. 2016. Health relevance of intestinal protein fermentation in young pigs. Anim. Health Res. Rev. 17(2):137–147. http://doi:10.1017/S1466252316000141 27572670 10.1017/S1466252316000141

[skaf451-B58] Pieper R. , Kröger S., Richter J. F., Wang J., Martin L., Bindelle J., Htoo J. K., von Smolinski D., Vahjen W., Zentek J. et al. 2012. Fermentable fiber ameliorates fermentable protein-induced changes in microbial ecology, but not the mucosal response, in the colon of piglets. J. Nutr. 142(4):661–667. http://doi:10.3945/jn.111.156190 22357743 10.3945/jn.111.156190

[skaf451-B59] Radlinski L. C. , Rogers A. W., Bechtold L., Masson H. L., Nguyen H., Larabi A. B., Tiffany C. R., Carvalho T. P. D., Tsolis R. M., Bäumler A. J. 2024. *Salmonella* virulence factors induce amino acid malabsorption in the ileum to promote ecosystem invasion of the large intestine. Proc. Natl. Acad. Sci. U S A. 121(47):e2417232121. http://doi:10.1073/pnas.2417232121 39546570 10.1073/pnas.2417232121 PMC11588050

[skaf451-B60] Renaud D. L. , Buss L., Wilms J. N., Steele M. A. 2020. Technical note: is fecal consistency scoring an accurate measure of fecal dry matter in dairy calves? J. Dairy Sci. 103(11):10709–10714. http://doi:10.3168/jds.2020-18907 32921450 10.3168/jds.2020-18907

[skaf451-B3749129] Rhine E. D. , Mulvaney R. L., Pratt E. J., and Sims G. K. 1998. Improving the berthelot reaction for determining ammonium in soil extracts and water. Soil Science Soc. of Amer. J. 62(2):473–480. 10.2136/sssaj1998.03615995006200020026x

[skaf451-B62] Rhouma M. , Fairbrother J. M., Thériault W., Beaudry F., Bergeron N., Laurent-Lewandowski S., Letellier A. 2017b. The fecal presence of enterotoxin and F4 genes as an indicator of efficacy of treatment with colistin sulfate in pigs. BMC Microbiol. 17(1):6–7. http://doi:10.1186/s12866-016-0915-0 28056796 10.1186/s12866-016-0915-0 PMC5217267

[skaf451-B63] Rocha G. C. , Duarte M. E., Kim S. W. 2022. Advances, implications, and limitations of low-crude-protein diets in pig production. Animals. 12(24):3478. http://doi:10.3390/ani12243478 36552397 10.3390/ani12243478 PMC9774321

[skaf451-B64] Rodrigues L. A. , Panisson J. C., Van Kessel A. G., Columbus D. A. 2022. Functional amino acid supplementation attenuates the negative effects of plant-based nursery diets on the response of pigs to a subsequent *Salmonella* typhimurium challenge. J. Anim. Sci. 100(10): skac267. http://doi:10.1093/jas/skac267 35976068 10.1093/jas/skac267 PMC9584161

[skaf451-B65] Rodrigues L. A. , Wellington M. O., González-Vega J. C., Htoo J. K., Van Kessel A. G., Columbus D. A. 2021. Functional amino acid supplementation, regardless of dietary protein content, improves growth performance and immune status of weaned pigs challenged with *Salmonella* typhimurium. J. Anim. Sci. 99(2):skaa365. http://doi:10.1093/jas/skaa365 10.1093/jas/skaa365 PMC8631066 33529342

[skaf451-B66] Soliani L. , Rugna G., Prosperi A., Chiapponi C., Luppi A. 2023. Salmonella infection in pigs: Disease, prevalence, and a link between swine and human health. Pathogens. 12(10):1267. http://doi:10.3390/pathogens12101267 37887782 10.3390/pathogens12101267 PMC10610219

[skaf451-B67] Song Y. , Mehl F., Zeichner S. L. 2024. Vaccine strategies to elicit mucosal immunity. Vaccines. (Basel). 12(2):191. http://doi:10.3390/vaccines12020191 38400174 10.3390/vaccines12020191 PMC10892965

[skaf451-B68] Snelson M. , Clarke R. E., Nguyen T. V., Penfold S. A., Forbes J. M., Tan S. M., Coughlan M. T. 2021. Long term high protein diet feeding alters the microbiome and increases intestinal permeability, systemic inflammation and kidney injury in mice. Mol. Nutr. Food Res. 65(8):2000851. http://doi:10.1002/mnfr.202000851 10.1002/mnfr.202000851 33547877

[skaf451-B69] Sung J. Y. , Adeola O. 2025. Increasing dietary indigestible protein may exacerbate coccidiosis in broiler chickens. Anim. Nutr. 22:13–18. http://doi:10.1016/j.aninu.2025.01.014 40808933 10.1016/j.aninu.2025.01.014 PMC12345305

[skaf451-B70] Tanaka T. , Imai Y., Kumagae N., Sato S. 2010. The effect of feeding lactic acid to *Salmonella* typhimurium experimentally infected swine. J. Vet. Med. Sci. 72(7):827–831. 20145379 10.1292/jvms.09-0490

[skaf451-B71] Tossou M. C. B. , Liu H., Bai M., Chen S., Cai Y., Duraipandiyan V., Liu H., Adebowale T. O., Al-Dhabi N. A., Long L. et al. 2016. Effect of high dietary tryptophan on intestinal morphology and tight junction protein of weaned pig. BioMed Res. Int. 2016(1):2912418. http://doi:10.1155/2016/2912418 27366740 10.1155/2016/2912418 PMC4913049

[skaf451-B72] Upadhaya S. D. , Kim I. H. 2021. The impact of weaning stress on gut health and the mechanistic aspects of several feed additives contributing to improved gut health function in weanling piglets—a review. Animals. 11(8):2418. http://doi:10.3390/ani11082418 34438875 10.3390/ani11082418 PMC8388735

[skaf451-B73] Verdonck F. , Cox E., van Gog K., Van der Stede Y., Duchateau L., Deprez P., Goddeeris B. M. 2002. Different kinetic of antibody responses following infection of newly weaned pigs with an F4 enterotoxigenic *Escherichia coli* strain or an F18 verotoxigenic *Escherichia coli* strain. Vaccine. 20(23–24):2995–3004. http://doi:10.1016/S0264-410X(02)00220-7 12126913 10.1016/s0264-410x(02)00220-7

[skaf451-B74] Wellington M. O. , Agyekum A. K., Hamonic K., Htoo J. K., van Kessel A. G., Columbus D. A. 2019. Effect of supplemental threonine above requirement on growth performance of *Salmonella* typhimurium challenged pigs fed high-fiber diets. J. Anim. Sci. 97(9):3636–3647. http://doi:10.1093/jas/skz225 31260524 10.1093/jas/skz225 PMC6735783

[skaf451-B75] Wellington M. O. , Thiessen R. B., Van Kessel A. G., Columbus D. A. 2020. Intestinal health and threonine requirement of growing pigs fed diets containing high dietary fibre and fermentable protein. Animals. 10(11):2055. http://doi:10.3390/ani10112055 33171958 10.3390/ani10112055 PMC7694666

[skaf451-B78] Won Y. K. , Kim S. J., Han J. H. 2021. The protective effect of dietary supplementation of *Salmonella*-specific bacteriophages in post-weaning piglets challenged with *Salmonella* typhimurium. J. Adv. Vet. Anim. Res. 8(3):440–447. http://doi:10.5455/javar.2021.h532 34722742 10.5455/javar.2021.h532 PMC8520144

[skaf451-B79] Xia J. , Fan H., Yang J., Song T., Pang L., Deng H., Ren Z., Deng J. 2022. Research progress on diarrhoea and its mechanism in weaned piglets fed a high‐protein diet. J. Anim. Physiol. Anim. Nutr. (Berl). 106(6):1277–1287. http://doi:10.1111/jpn.13654 34719816 10.1111/jpn.13654

[skaf451-B80] Xu J. , Jia Z., Xiao S., Long C., Wang L. 2023. Effects of enterotoxigenic *Escherichia coli* challenge on jejunal morphology and microbial community profiles in weaned crossbred piglets. Microorganisms. 11(11):2646. http://doi:10.3390/microorganisms11112646 38004658 10.3390/microorganisms11112646 PMC10672776

[skaf451-B81] Yi G. F. , Carroll J. A., Allee G. L., Gaines A. M., Kendall D. C., Usry J. L., Toride Y., Izuru S. 2005. Effect of glutamine and spray-dried plasma on growth performance, small intestinal morphology, and immune responses of *Escherichia coli* K88+-challenged weaned pigs. J. Anim. Sci. 83(3):634–643. http://doi:10.2527/2005.833634x 15705760 10.2527/2005.833634x

[skaf451-B82] Yin F. , Farzan A., Wang Q., Yu H., Yin Y., Hou Y., Friendship R., Gong J. 2014. Reduction of *Salmonella enterica* serovar typhimurium DT104 infection in experimentally challenged weaned pigs fed a lactobacillus-fermented feed. Foodborne Pathog. Dis. 11(8):628–634. http://doi:10.1089/fpd.2013.1676 24826965 10.1089/fpd.2013.1676

[skaf451-B84] Zhang H. , van der Wielen N., van der Hee B., Wang J., Hendriks W., Gilbert M. 2020. Impact of fermentable protein, by feeding high protein diets, on microbial composition, microbial catabolic activity, gut health and beyond in pigs. Microorganisms. 8(11):1735. http://doi:10.3390/microorganisms811173 33167470 10.3390/microorganisms8111735 PMC7694525

[skaf451-B85] Zhang L , Piao X. 2022. Different dietary protein sources influence growth performance, antioxidant capacity, immunity, fecal microbiota and metabolites in weaned piglets. Anim. Nutr. 8(1):71–81. http://doi:10.1016/j.aninu.2021.06.013 34977377 10.1016/j.aninu.2021.06.013 PMC8669252

[skaf451-B86] Zhang S. , Kingsley R. A., Santos R. L., Andrews-Polymenis H., Raffatellu M., Figueiredo J., Nunes J., Tsolis R. M., Adams L. G., Bäumler A. J. 2003. Molecular pathogenesis of *Salmonella* enterica serotype typhimurium-induced diarrhea. Infect. Immun. 71(1):1–12. http://doi:10.1128/IAI.71.1.1-12.2003 12496143 10.1128/IAI.71.1.1-12.2003 PMC143292

[skaf451-B87] Zhao X. , Liu Y., Ding H., Huang P., Yin Y., Deng J., Kong X. 2021. Effects of different dietary protein levels on the growth performance, serum biochemical parameters, fecal nitrogen, and carcass traits of Huanjiang mini-pigs. Front. Vet. Sci. 8:777671. http://doi:10.3389/fvets.2021.777671 34988141 10.3389/fvets.2021.777671 PMC8720777

